# Optimization of a Solution-Processed TiO_*x*_/(n)c-Si Electron-Selective Interface by Pre- and Postdeposition Treatments

**DOI:** 10.1021/acsami.3c18134

**Published:** 2024-03-19

**Authors:** Naser Beyraghi, Mehmet C. Sahiner, Oguzhan Oguz, Selcuk Yerci

**Affiliations:** † ODTU-GUNAM, Middle East Technical University, Ankara 06800, Turkey; ‡ Department of Micro and Nanotechnology, Middle East Technical University, Ankara 06800, Turkey; § Department of Electrical and Electronics Engineering, Middle East Technical University, Ankara 06800, Turkey

**Keywords:** titanium oxide, solution-processed, low temperature, silicon surface passivation, passivation mechanism, electron-selective heterocontact

## Abstract

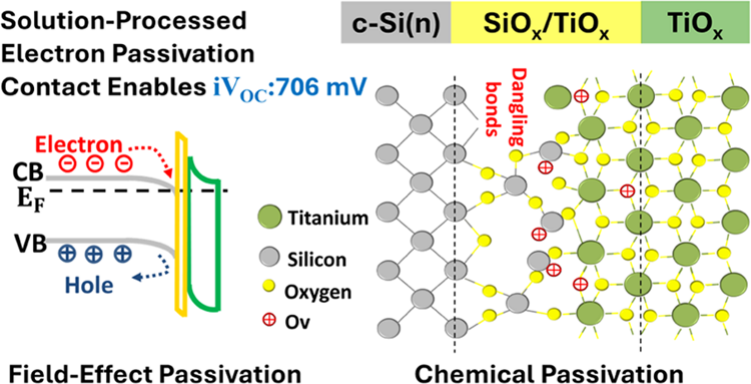

Developing a vacuum-free and low-temperature deposition technique for dopant-free carrier-selective materials without sacrificing their performance can reduce the fabrication cost and CO_2_ footprint of silicon heterojunction (SHJ) solar cells. In this contribution, to activate the full capacity of the solution-processed TiO_*x*_ as an electron-selective passivation contact, the effects of various pre- and postdeposition treatments on the passivation quality and contact resistivity are investigated simultaneously. It is demonstrated that the electrical properties of a thin TiO_*x*_ layer spin-coated on an n-type silicon substrate can be remarkably improved through tailor-made pre- and postdeposition treatments. A notable low surface recombination velocity (SRV) of 6.54 cm/s and a high implied open-circuit voltage (i*V*_oc_) of 706 mV are achieved. In addition, by inserting a 1 nm LiF_*x*_ buffer layer between TiO_*x*_ and Al metal contact, a low contact resistivity (ρc) of 15.4 mΩ·cm^2^ is extracted at the n-Si/SiO_*x*_/TiO_*x*_ heterojunction. Our results bring the solution-processed TiO_*x*_ electrical properties to a level on par with those of state-of-the-art pure TiO_*x*_ layers deposited by other techniques. Chemical and electrical characterizations elucidate that the improved electrical properties of the investigated Si/SiO_*x*_/TiO_*x*_ heterojunction are mediated by the concomitant involvement of chemical and field-effect passivation.

## 1 Introduction

The concept of carrier-selective contacts (CSCs) has emerged and been thoroughly established during the last decade to circumvent the detrimental features of direct metal-to-silicon absorber contact and/or the heavily diffused regions. In principle, the main objectives of CSCs are isolating direct contact of semiconductor and metal, thereby minimizing the surface recombination velocity and also extracting only one type of carrier (electron or hole) from the relevant contact with low resistive losses. These objectives, observed significantly by the state-of-the-art CSC schemes, rely on the stack of a thin passivation dielectric layer (i.e., hydrogenated intrinsic amorphous silicon (i)a-Si:H or SiO_2_) and a doped (p or n) amorphous or polycrystalline silicon selective layer, integrated into ultra-high-efficiency silicon heterojunction solar cells (>25%).^1−5^ Nevertheless, due to the doping process incorporation in the silicon-based CSCs, inherent issues, such as small optical band gap and parasitic photon absorption, still hinder the further improvement of the cell’s performance. In addition, the fabrication of Si-based CSCs is carried out in the relatively expensive vacuum-based plasma-enhanced chemical vapor deposition (PECVD) system, where toxic compounds of boron/phosphorus gas precursors are used for doping and the existence of high-security control systems is inevitable. Furthermore, the doping process of polycrystalline silicon is accompanied by a high-temperature budget for dopant diffusion and curing purposes.^6−9^ These requirements make the fabrication process of Si-based CSCs more complex and expensive, giving rise to the cost-to-watt ratio. Therefore, the quest to address the issues associated with doped Si-based CSCs brought increasing attention to developing high-efficiency dopant-free counterparts compatible with simple fabrication techniques. The concept of CSCs expanded to include wide band gap dopant-free carrier-selective alternatives, such as transition metal oxides (TMOs), fluorides, and organic materials, where inspiring surface passivation quality and carrier selectivity with low resistive losses could be successfully delivered.^10−16^ The electron selectivity characteristic of thin titanium oxide (TiO_*x*_, with *x* ≤ 2)-based heterojunction has been proven in numerous works, making it one of the most promising n-type CSC alternatives.^17−22^ In addition to the excellent chemical surface passivation of an amorphous TiO_*x*_ layer on a silicon substrate, on account of its low work function (WF ∼ 4 eV) and wide band gap (∼3–3.5 eV), it creates a small conduction band offset (∼0.05 eV) and a large valence band offset (∼2 eV) at the Si/TiO_*x*_ interface, allowing electrons to pass through the junction while blocking hole transportation.^6,7,9^ To date, to derive the utmost performance and the least fabrication cost of TiO_*x*_-based CSCs, various deposition techniques and pre- and postdeposition treatments have been examined. Different low-temperature deposition techniques, such as atomic layer deposition (ALD),^17−23^ chemical vapor deposition (CVD),^24,25^ e-beam evaporation,^26,27^ spray and spin coating,^28,29^ have been employed. Prior to TiO_*x*_ deposition, the insertion of an ultrathin tunnel oxide layer between the substrate and TiO_*x*_ has been suggested to enhance the passivation quality by oxygen termination of dangling bonds on the silicon surface. In this respect, various wet-chemical (RCA2, H_2_O_2_, HNO_3_, ozonized DI-H_2_O), dry photo UV/O_3_, and thermal oxidation methods have been explored to synthesize such an oxide layer.^11,30−33^ Moreover, the prominence of postdeposition treatments, such as hydrogen plasma,^34,35^ light soaking,^12,36^ and particularly thermal activation,^7,27^ on the TiO_*x*_ electrical properties has been extensively investigated. The solution-process-based methods hold advantages over other deposition techniques in terms of simplicity and fabrication cost, allowing thin films to be readily deposited at room temperature and atmospheric pressure. Despite these merits, only a few studies evaluated and discussed the properties of solution-processed TiO_*x*_. Some of these works focused on the optical and electrical properties of a crystalline thick TiO_2_ (over 40 nm) layer, which is a well-suited material for antireflection coating (ARC) application as it has a large refractive index and band gap.^28,29,37^ Benefiting from a 60 nm TiO_*x*_ layer on an n-type silicon substrate, Sun et al. reported a high i*V*_oc_ and an effective lifetime of 695 mV and 1.11 ms, respectively.^38^ Nevertheless, such a thick layer cannot be employed in the CSC structures, where ideally an amorphous thin layer of TiO_*x*_ is in demand. Li et al. exploited a (i)a-Si:H/SiO*_x_*/TiO_2_ stack as CSC integrated into a dopant-free interdigitated back-contact silicon heterojunction (IBC-SHJ) solar cell with an efficiency of 20.24%.^33^ Although TiO_*x*_ was manufactured by the spin-coating method, the observed efficiency could not be attained without a (i)a-Si:H interfacial passivation layer, which necessitates the use of the vacuum-based PECVD technique. Lee et al. presented a high surface recombination velocity (SRV) of 625 cm/s with an 8 nm TiO_*x*_ layer reflected in a V_oc_ of 600 mV at the cell level with a front side passivated by a a-Si:H(i)/a-Si:H(p) stack.^39^ In fact, the current state-of-the-art ALD-deposited TiO_*x*_ substantially outperforms the reported solution-processed TiO_*x*_-based CSCs; thus, further progress has yet to be made to achieve a competitive level of performance. In this context, this work studies the effects of predeposition termination of silicon surface dangling bonds as well as postdeposition annealing atmospheres to discover viable approaches for optimizing the electrical properties of the Si/TiO_*x*_ interface. Implementing an RCA2 growth SiO*_x_*/spin-coated TiO_*x*_ stack and a low thermal budget-annealing step, a high level of silicon surface passivation coupled with a low contact resistivity is achieved. Our findings bring the solution-processed TiO_*x*_ electrical properties to a level on par with state-of-the-art pure TiO_*x*_ layers deposited by other techniques. We demonstrated that the enhanced electrical properties of the Si/SiO*_x_*/TiO_*x*_ interface are provided by a combination of chemical and field-effect passivation. Analyses carried out here justify the interaction among Si, O, and Ti atoms, the presence of oxygen vacancies (Ov), and Ti lower oxidation states at the interface, which are promoted in the course of thermal activation.

## 2 Materials and Methods

### 2.1 Sample Preparation and Characterization

To prepare the TiO_*x*_ solution, first, TDIP, the precursor solution (0.2 mL, titanium diisopropoxide bis(acetylacetonate), 75% in isopropanol, from Sigma-Aldrich) was mixed with 1-butanol (4.3 mL, 99.9%, from Sigma-Aldrich). Then, the dilute hydrochloric acid (2M-10 μL) and DIW (30 μL) were added dropwise to the stirring mixture and kept stirring for at least 1 h. As shown in Figure S1 (Supporting Information), to produce TiO_*x*_ films with different thicknesses, this mixture was diluted with additional 1-butanol at different volume ratios and kept on a stirring plate overnight.

In this work, 4-in. round double-side polished n-type <100> oriented float-zone (FZ) crystalline silicon wafers with a resistivity of 1–3 Ω·cm and a thickness of 280 μm were used as substrates for passivation studies, unless otherwise stated. Before TiO_*x*_ coating, three groups of silicon surfaces were prepared via different pretreatments. To do so, at first, the round wafers were cut into quarter pieces and went through the standard sequence of RCA1 and RCA2 cleaning procedures to terminate the silicon surface with RCA2 oxide. Afterward, on a part of the samples, the RCA2-grown oxide was kept, while for the rest, it was removed by a short immersion in the dilute hydrofluoric acid (HF), resulting in a hydrogen-terminated silicon surface. For photogenerated dry oxide synthesis, a part of the hydrogen-terminated samples was symmetrically exposed to UV irradiation for 10 min. UV/O_3_ oxidation was carried out at room temperature in a Novascan PSD Series UV Ozone Cleaner. The TiO_*x*_ solution was then spin-coated on both sides of the silicon substrates at 3000 rpm for 30 s to fabricate the TiO_*x*_ thin film that will be referred to as the as-deposited TiO_*x*_, which was subsequently subjected to an annealing step at either nitrogen (N_2_, 99.999%), forming gas (97% N_2_ and 3% H_2_) within a quartz tube furnace, or on a hot plate in ambient air for thermal activation.

The thickness study was carried out by spectroscopic ellipsometry (SE), and the Tauc–Lorentz dispersion model was utilized to fit the delta and psi spectra. i*V*_oc_ and τ_eff_ values were extracted at 1 sun injection density and characterized by the quasi-steady state photoconductance (QSSPC) method using a WCT-120 Sinton instrument. Accordingly, the upper limit of the equivalent surface recombination velocity (SRV) was calculated using eq 1. The surface recombination current density (*J*_0_) values were calculated from the slope of the linear fit to the inverse lifetime data at high injection conditions (i.e., Δ*n* ≫ *N*_dop_) based on the equations shown below:
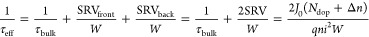
1
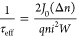
2where *W*, *n*_i_, and *N*_dop_ indicate the substrate thickness, intrinsic carrier concentration in silicon, and background dopant density, respectively, in eq 1. Considering the symmetrical structure of passivation samples (both sides coated with TiO_*x*_), the SRV_front_ and SRV_back_ are equal. In addition, since FZ wafers were used as the substrate, the bulk lifetime (τ_bulk_) can be assumed to be infinite. Thus, at a high injection level, the inverse lifetime can be written as in eq 2. Note that the reported *J*_0_ is the double-side *J*_0_ of the symmetric passivation test samples and is not divided by two. Contact resistivity values were extracted by the transfer-length-method (TLM). Metal strips (Al: 200 nm, LiF_*x*_/Al: 1:200 nm) with a length and width of 1 and 0.05 cm, respectively, were thermally evaporated through a shadow mask with pad spacing starting from 500 to 1300 μm. Surface topography of the samples was explored with atomic force microscopy (AFM) in tapping scanning mode via Nanomagnetics instruments. Attenuated total reflectance Fourier transform infrared (ATR-FTIR) and transmission FTIR measurements were done using a Nicolet iS50 FTIR spectrometer of Thermo Scientific and an Agilent Cary 630 FTIR spectrometer, respectively. Photoluminescence images were taken by a SEMILAB (PLI-1001) setup, which is equipped with a quasi-steady-state microwave photoconductance decay (QSS-μPCD) system, enabling lifetime extraction. X-ray photoelectron spectroscopy (XPS) experiments were carried out (Physical Electronics (PHI), Versa Probe 5000) under a high vacuum with a base pressure of 1 × 10^–9^ mbar. An X-ray energy of 22.9 W, a pass energy of 58.70 eV, an energy step of 0.1 eV, an X-ray spot size of 100 μm, and an angle of 45° were used. All peaks were calibrated with the carbon 1s peak at 284.7 eV, and the peaks were fitted with Gaussian–Lorentzian curves. Current–voltage (*C*–*V*) measurements were conducted on an MFIA impedance analyzer by Zurich Instruments with samples enclosed in a probe station utilizing a Faraday cage architecture built in-house under dark conditions at room temperature. Capacitance–voltage traces were recorded using a small amplitude sinusoidal perturbation of a 15 mV peak at 1 MHz superimposed on a DC bias starting from inversion and cyclically swept into the accumulation and back to inversion with a sweep rate of 0.1 V/s. Contact potential difference (CPD) measurements were made with an Oxford instrument (MFP-3D origins) device. A Co–C tip with a work function (WF) of 4.9 eV was used to probe the surface of the samples placed on a grounded stage. A gold-coated silicon sample was used as a reference to extract the WF through the following equation:
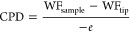
3

## 3 Results and Discussion

### 3.1 Surface Passivation and Contact Resistivity Optimization

**Figure 1:**
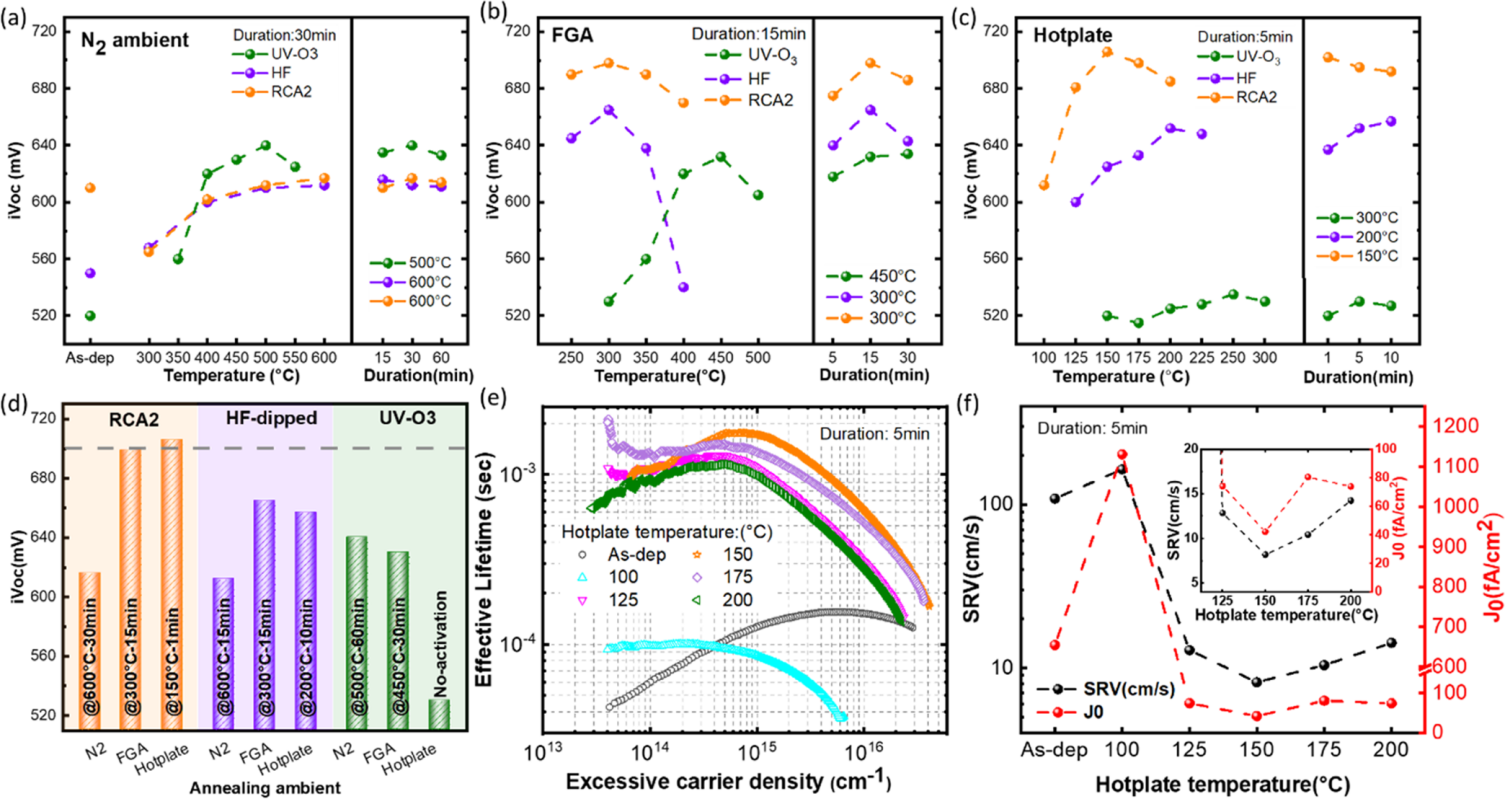
Measured i*V*_oc_ as a function of annealing temperature and duration at (a) N_2_, (b) FGA, and (c) room ambience; (d) maximum i*V*_oc_ for different combinations of pre- and post-treatments obtained at the optimal annealing temperature and duration. (e) Injection-level-dependent effective lifetime and (f) the upper limits of surface recombination velocity (SRV) and surface recombination current density (*J*_0_) of the passivation samples produced on the RCA2-treated substrates and annealed at different hot plate annealing temperatures for fixed 5 min (the inset provides a clear view of the obtained SRV and *J*_0_ values for the annealing temperatures higher than 125 °C). The dashed lines on panels (a), (b), (c), and (e) are provided to guide the eye.

The thickness of carrier-selective contact plays a crucial role in determining its electrical properties; on the one hand, it must be thick enough to effectively cover the surface and suppress the surface recombination losses; on the other hand, since the conductivity of CSC is restricted by its semiconducting or insulating nature, its thickness must be thin enough to minimize resistance against carrier transportation. It has been demonstrated that with a thickness in the range of 2–10 nm, TiO_*x*_ can sufficiently afford surface passivation and electron extraction simultaneously.^9,33,43^ The volume ratio of chemicals used in the solution preparation is tailored to set the spin-coated TiO_*x*_ film thickness to the desired range. With the described recipe (Materials and Methods section) and a dilution ratio of 1:3 (TiO_*x*_ solution/1-butanol solvent), a thin film down to 7.1 nm in thickness is achieved. Figure 1 depicts the surface passivation quality of samples subjected to distinct pre- and postdeposition treatments. The measured i*V*_oc_ was plotted versus the annealing temperature and duration to identify the optimal passivation level for each combination. As shown in Figure 1a, the as-deposited TiO_*x*_ exhibits a relatively low i*V*_oc_ of 610, 551, and 519 mV on the RCA2, HF-dipped, and UV–O_3_-pretreated samples, respectively. Post-thermal activation was carried out in different ambiences to enhance the passivation quality. In a N_2_ atmosphere, for RCA2 and HF-dipped samples, the maximum i*V*_oc_ of 617 and 611 mV was obtained at 600 °C, respectively. The UV–O_3_ sample exhibits the largest improvement, where its i*V*_oc_ increases to 640 mV after 30 min of annealing at 500 °C. At these optimum temperatures, variations in annealing duration have an insignificant effect on passivation quality. Despite a 121 mV increase in i*V*_oc_ for the UV–O_3_ sample, the passivation quality in N_2_ annealing is inadequate to achieve high-performance solar cells. Figure 1b illustrates the influence of annealing at the forming gas atmosphere on the i*V*_oc_. FG annealing at 300 °C substantially reinforces the surface passivation quality of RCA2 and HF-dipped samples, accounting for an i*V*_oc_ of 698 and 665 mV, respectively. Annealing at temperatures higher than 300 °C deteriorates the passivation quality of both samples but more severely for the HF-dipped sample, revealing its inferior thermal stability, consistent with the observations in refs (6,7). In contrast, the i*V*_oc_ of the UV–O_3_ pretreated decreases to 630 mV after annealing at 450 °C, which is 10 mV lower than its maximum under N_2_ ambience. Annealing duration is the other parameter that significantly impacts the passivation quality in FGA. The maximum passivation activation occurs when the samples are annealed for 15 min at their respective optimum annealing temperatures. Figure 1c presents the variation in i*V*_oc_ as a function of hot plate annealing temperature and time carried out in room ambience with a relative humidity of ∼40–50%. For RCA2 and HF-dipped samples, there is a discernible correlation between hot plate annealing temperature and passivation quality, whereas the i*V*_oc_ of the UV–O_3_ sample demonstrates no significant dependence on the annealing conditions. When the annealing duration is 5 min, the i*V*_oc_ progressively increases with the annealing temperature increment, reaching the maximum i*V*_oc_ of 695 mV at 150 °C and 652 mV at 200 °C on RCA2 and HF-dipped samples, respectively. Annealing at these optimal temperatures for various durations reveals that the passivation of the HF-dipped sample improves slightly with extended annealing duration, reaching 657 mV after 10 min, while on the RCA2 sample, it improves at shorter annealing times, reaching the highest recorded value of 706 mV after 1 min of annealing. The reproducibility of high-level passivation under the proposed optimal conditions is validated in Figure S2a,b. The optimum annealing point of all combinations and the acquired maximum i*V*_oc_ of which have been summarized in Figure 1d. These results highlight the pivotal role of pre- and postdeposition treatments on the passivation quality of the investigated TiO_*x*_ layer. It is evident that excellent surface passivation close to 700 mV is feasible only in the presence of RCA2 oxide, which chemically deactivates recombination centers on the silicon surface. However, oxygen termination of dangling bonds on silicon surfaces does not necessarily promote passivation performance; for instance, the hydrogen-terminated sample annealed in FGA and on the hot plate has higher i*V*_oc_ than that of the UV–O_3_-treated sample. Due to distinct oxide growth kinetics in RCA2 and UV–O_3_ methods, their chemical natures are different, which results in contrasting interfacial properties. In addition, we observe that except for hot plate annealing of the UV–O_3_ sample, annealing is an essential prerequisite for passivation activation of the as-deposited TiO_*x*_ layer. For thermal passivation activation of TiO_*x*_ coated on RCA2-treated samples, hot plate annealing in an ambient room and FGA are the optimal environments. The corresponding effective carrier lifetime (τ_eff_) of RCA2-pretreated samples presented in Figure 1c is displayed in Figures 1e and S2c as a function of the injection level. It is observed that the as-deposited sample has a prolonged lifetime at higher injection than at lower injection regions, while the annealed samples have a longer lifetime at lower injection regions. The conversion of lifetime behavior versus injection level after annealing could likely be caused by the emergence of a strong built-in potential field effect at the interface.^40^ At an injection level of 10^15^ cm^–3^, the τ_eff_ increases from 128.6 μs in the as-deposited state to 1713 μs after 5 min of hot plate annealing at 150 °C. Figure 1f presents the extracted SRV and *J*_0_ values of these samples. The SRV and *J*_0_ of 108.8 cm/s and 655 fA/cm^2^ in the as-deposited state dramatically diminish to 8.17 cm/s and 42.1 fA/cm^2^, respectively, by annealing at 150 °C, as illustrated in Figure 1f inset. Under optimal conditions, SRV and *J*_0_ experience further reduction, reaching 6.54 cm/s and 36.7 fA/cm^2^, respectively, as depicted in the legend of Figure S2c. With regard to passivation stability, it was noted that the high level of passivation quality provided under optimal conditions fades away by time as the samples are exposed to the room ambience; however, it is sustained when the samples are stored in a nitrogen box, as displayed in Figure S2d. Therefore, to retain the passivation quality, it is vital to fabricate solar cells immediately after annealing or to protect the samples in an oxygen- and water-free environment to avoid the interaction of oxygen and hydroxyl radicals with the TiO_*x*_ film.

**Figure 2:**
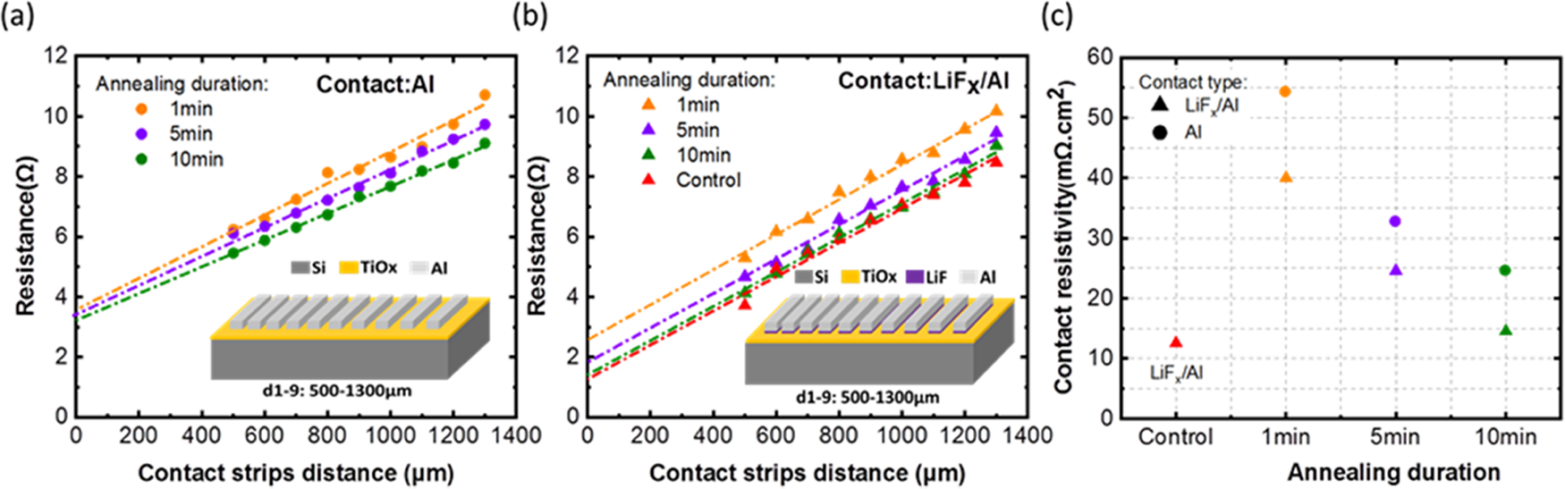
Resistance versus contact strip distance plot of TLM test structures with (a) Al and (b) LiF_*x*_/Al contact type (the insets sketch TiO_*x*_-coated TLM structures with Al and LiF_*x*_/Al contacts), and (c) extracted contact resistivity. (All samples are prepared on RCA2-pretreated CZ n-Si (1–3 Ω·cm, 280 μm) substrates, and annealing was carried out on a hot plate at 150 °C for 1, 5, and 10 min.)

Similar to high surface passivation, achieving a low contact resistivity (ρc) against electron extraction is of key importance for a functional electron-selective passivation contact. To assess ρc, the transfer-length-method (TLM) test structure was fabricated on n-type CZ silicon substrates that received RCA2 pretreatment. Following TiO_*x*_ deposition on one side of the test sample and annealing on the hot plate at 150 °C for various durations, Al or a stack of LiF_*x*_/Al was thermally evaporated through a shadow mask designed to define the contact geometry on the sample as schemed in Figure 2a,2b insets. Control test structures were prepared without a TiO_*x*_ layer for comparison. The LiF_*x*_/Al (LiF_*x*_ thickness: 1 nm) bilayer contact is employed to enhance the electron extraction capability of the investigated TiO_*x*_. It has been demonstrated that the use of low-work-function LiF_*x*_ lowers the band structure (Fermi level) of the Si surface, encouraging the collection of electrons.^41−43^ Both sides of the test sample were laser-cut before measurement to confine the lateral spreading contribution (Figure S3a). The dark current–voltage (*I*–*V*) curves of various test structures are presented in Figure S3b,c. All *I*–*V* curves, except the Al control sample, exhibit an ohmic behavior, making the ρc extraction feasible. The ρc values are extracted by extrapolation of resistance (calculated from IV curves) versus pad spacing, as illustrated in Figure 2a,b. The dependence of the ρc value on LiF_*x*_ insertion and annealing duration is given in Figure 2c. For the control sample with LiF_*x*_/Al contact, a relatively low ρc of 13.2 mΩ·cm^2^ was obtained. ρc decreases with the annealing duration, regardless of the contact type for TiO_*x*_-coated samples. The lowest ρc of 15.4 mΩ·cm^2^ was achieved for TiO_*x*_/LiF_*x*_/Al contact, which is slightly larger than that of the control structure and is in the range of the lowest contact resistances reported for TiO_*x*_-based heterojunctions. Considering the results illustrated in Figures 1c and 2c, a trade-off between passivation quality and ρc upon annealing duration is present. Longer annealing deteriorates the passivation quality but improves electron extraction. Providentially, even after 10 min of annealing, a high level of passivation (i.e., τ_eff_: 1191 μs, i*V*_oc_: 683 mV) can still be achieved. Table 1 compares the CSC features of TiO_*x*_ presented in this work with other TiO_*x*_-based heterocontacts reported in the literature. Our results bring the solution-processed TiO_*x*_ electrical properties to a level on par with state-of-the-art pure TiO_*x*_ layers deposited by other techniques, such as the advanced ALD technique, which provides pinhole-free films with perfect control over the deposited layer thickness, manifesting the high and comparable potential of the solution-processed TiO_*x*_. A higher τ_eff_ (4000 and 3030 μs) was reported in refs (12,44) than our work; however, the implemented 60 nm layer is too thick and not appropriate for electron extraction and was indeed tailored to be used as an ARC layer.^12^ Furthermore, Wang et al. utilized thicker silicon substrates with higher base resistivity, which restricts the recombination at bulk and surface defect sites and enhances the extracted τ_eff_.^44^ We would like to make two points clear: first, since the doping is out of our scope in this work, only the results of single-layer, undoped TiO_*x*_ are included and doped TiO_*x*_ results are not compared^22,45−47^ in Table 1. Second, it must be noted that all of the passivation parameters reported in Table 1 are for prior metallization. It has been demonstrated that the adverse influence of metallization (Al) on the passivation quality is a common concern for all TiO_*x*_-based contacts, which imposes a reduced *V*_oc_ compared with i*V*_oc_ at the cell level.^49−51^

**Table 1:** Summary of TiO*_x_*-Based (with *x* ≤ 2) CSC Electrical Properties Fabricated with Different Techniques and Processing Conditions

ref #	substrate type	substrate thickness (μm)	substrate resistivity (Ω·cm)	deposition technique	TiO*_x_*/TiO_2_ thickness (nm)	annealing condition	contact type	analyze mode	ρ_c_ (mΩ·cm^2^)	i*V*_oc_ (mV)	lifetime (μs)	*J*_0_ (fA/cm^2^)	SRV (cm/s)
(6)	n-FZ	175	1	ALD	4.5	FG@250 °C-3 min	Al/Ag	QSSPC	20		850		11
(7)	n-FZ	175	1	ALD	5.5	FG@250 °C-30 min	Al/Ag	QSSPC	250	703	865	25	10
(8)	n-FZ	155	0.9	ALD	3.5	FG@250 °C-5 min	Ca/Al	QSSPC	5		800	50	15
(12)	n-FZ	280	2.5	ALD	60	N_2_@250 °C-30 min		QSSPC			4000		2.8
(20)	n-FZ	180	1	ALD	15			QSSPC				10	2.5
(36)	n-FZ		5	APCDV	60	N_2_@250 °C-30 min		QSSPC			730		30
(9)	n		1.7	E-beam	2	O_2_@250 °C-10 min	LiF/Al	QSSPC	106	672		100	13.7
(38)	n-FZ	280	2	spin-coat	60			QSSPC		695	1110	26.7	6.25
(11)	n-FZ	280	2–5	ALD	4	FG@350 °C-3 min			25		1473	20	9.2
(26)	n-FZ	300	2–5	E-beam	2	O_2_@250 °C-10 min	Al	QSSPC	120	660	750		15
(44)	n-FZ	400	5	ALD	4	N_2_@250 °C-30 min	LiF/Al	QSSPC	18		3030	23	
(48)	n-FZ	280	2–5	ALD	4.5	FG@400 °C-3 min					891		16
(43)	n-CZ	270	1–3	ALD	5.5		LiF/Al	QSSPC	20				10
(27)	n-FZ	500	8	E-beam	3.5	O_2_@250 °C-10 min		QSSPC			1500		16
(33)	n-CZ	220	1–5	spin-coat	10	Hot plate@140 °C-5 min	Mg/Al/Ag	QSSPC			<1000	13.1	
this work	n-FZ	280	2.7	spin-coat	7.1	Hot plate@150 °C-10 min	LiF/Al	QSSPC	15.4	683	1191	81.4	11.75
this work	n-FZ	280	2.7	spin-coat	7.1	Hot plate@150 °C-1 min	Al	QSSPC	55	706	2140	36.7	6.54

### 3.2 Characterization

To acquire a comprehensive insight into the underlying mechanism of the remarkable surface passivation and electron selectivity features of the TiO_*x*_ fabricated under optimal pre- and postdeposition conditions, different structural, chemical, and electrical characterizations were conducted. It is well established that amorphous TiO_*x*_ provides lower interface defect density (*D*_it_) and superior surface passivation quality, while the evolution toward crystalline phases caused by annealing enlarges the lattice mismatch between the crystalline TiO_*x*_ film and the c-Si substrate, introducing high *D*_it_ and severely devastating its passivation feature.^6−8,12,18^ As noted in Figure 1c, the passivation quality deteriorates either with an extended annealing duration at 150 °C or with higher annealing temperatures. Crystallization and grain boundary formation are reflected by an increase in the surface roughness, which could be characterized by atomic force microscopy (AFM) measurements.^17,34,48^ The three-dimensional (3D) surface morphology of samples prepared under various conditions is displayed in Figure S4a. It reveals that elevating the annealing temperature and extending the annealing duration roughen the surface. Considering the fact that the onset of TiO_*x*_ crystallization occurs at temperatures above 300 °C, consequently, the passivation degradation here is more likely due to the rupture in the TiO_*x*_ layer coverage rather than crystallization. Nevertheless, the structural properties of the TiO_*x*_ film cannot fully describe its passivation behavior, and for an in-depth comprehension, the interface chemical and electrical natures modified by the annealing step must be taken into account as well. In previous reports, the passivation property of TiO_*x*_ has been commonly correlated with the interaction between Si, O, and Ti atoms; hence, the presence of bindings, such as Si–O–Si and Si–O–Ti between these atoms, is conceded as evidence of TiO_*x*_ chemical passivation.^24,26,28,44^ Herein, through attenuated total reflectance Fourier transform infrared (ATR-FTIR) and transmission FTIR, the chemical properties of the TiO_*x*_ solution and the spin-coated TiO_*x*_ film on silicon substrates have been investigated. The chemical structures and full absorption spectrum of the solvents composing the TiO_*x*_ solution were individually illustrated in Figure S5a, indicating that the TiO_*x*_ solution is primarily composed of methyl groups and acetate ligands linked to Ti–O and hydroxyl groups. In the fingerprint region (i.e., below 1400 cm^–1^, Figure S5b), numerous characteristic vibrational modes of different Si, O, and Ti interactions have been detected; for instance, peaks at 1028 and 1080 cm^–1^ correspond to Si–O stretching vibration of substoichiometric SiO_*x*_ and stoichiometric SiO_2_, respectively.^52^ The peak appearing at 667 cm^–1^ is associated with Ti–O bond vibration, while the Ti–O–Si vibration is located at 930 cm^–1^.^38,53^ The observation of Si–O, Ti–O, and particularly Ti–O–Si vibration modes suggests that the incorporation of a TiO_*x*_ layer on the silicon surface drives chemical reactions at the Si/SiO_*x*_/TiO_*x*_ interface.

**Figure 3:**
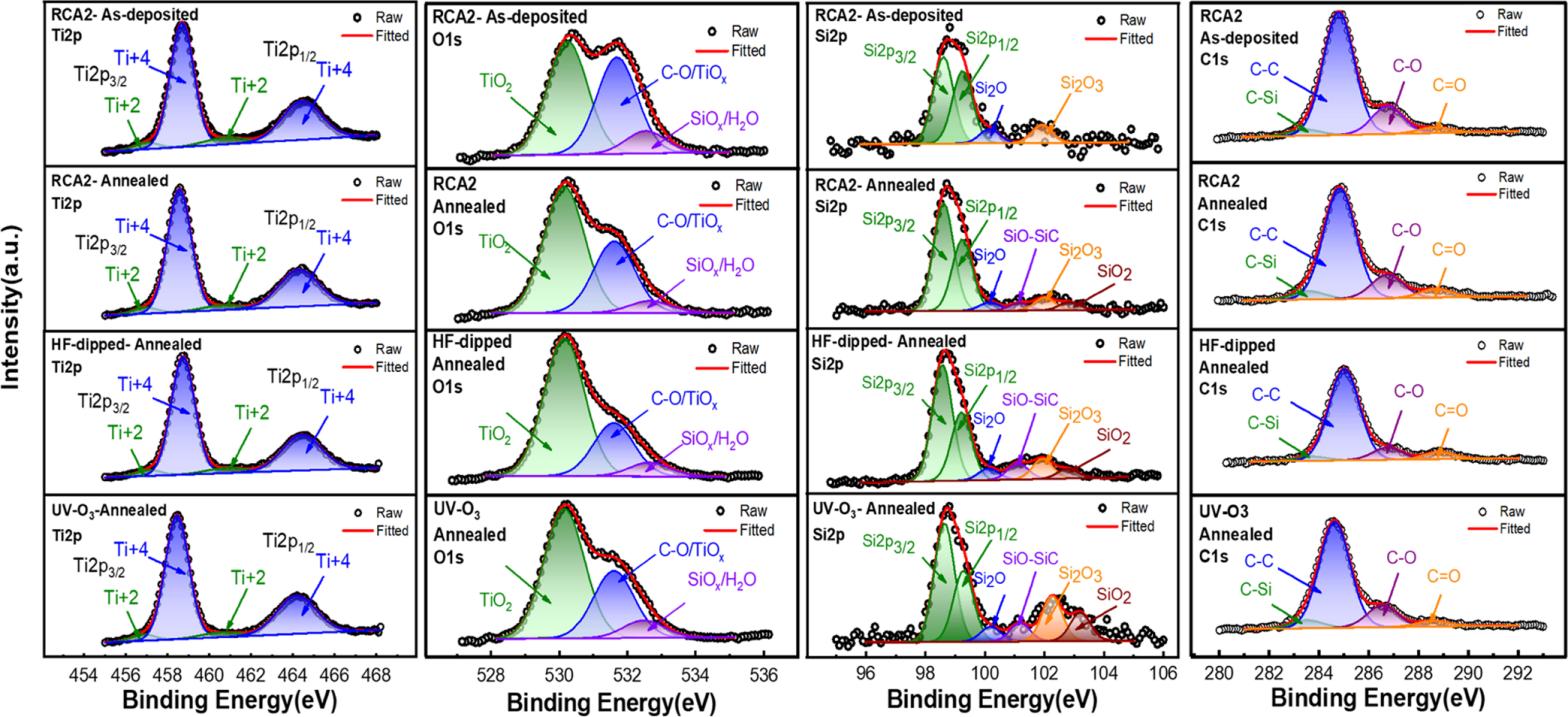
XPS Ti 2p, O 1s, Si 2p, and C 1s core-level spectra (from left to right) of the TiO_*x*_ film coated on RCA2, HF-dipped, and UV–O_3_-pretreated substrates. Note that the first and second rows compare the associated spectra of the TiO_*x*_ film coated on the RCA2-pretreated substrate before and after annealing. (Refer to Table S2 for specifics on the preparation conditions of XPS sample.)

X-ray photoelectron spectroscopy (XPS) was used to study the chemical composition and oxidation state of the samples (as described in Table S2) coated with solution-processed TiO_*x*_. The survey scan identified peaks associated with Ti, O, Si, and C elemental species (Figure S6a). The high-resolution XPS core-level spectra of Ti 2p, O 1s, Si 2p, and C 1s for as-deposited and annealed TiO_*x*_ on silicon surfaces received different pretreatments are shown in Figure 3 (from left to right), respectively. The Ti 2p spectrum can be fitted with two doublets: the first doublet exhibits two peaks at 456.92 and 460.59 eV, while the second doublet possesses peaks at 458.56 and 464.28 eV, which are assigned to the 2p_3/2_ and 2p_1/2_ electron spin orbitals of Ti^2+^ (in TiO_*x*_) and Ti^4+^ (in TiO_2_), respectively.^54^ The O 1s spectrum was deconvoluted into three singlets with peak positions of 530.1, 531.5, and 532.6 eV, attributed to the lattice oxygen (O^2–^) of Ti^4+^, the oxygen (O^–^) close to oxygen vacancy or coupled with –C, and the SiO_*x*_ /hydroxyl group, respectively.^38,44,55^ The percentage of each component detected in different spectra is given by the ratio between the integrated area under a particular peak and the total area under peaks (Figure S6b) (Note: These percentages refer to regions close to the TiO_*x*_ surface). The variation in Ti^4+^ and Ti^2+^ percentages in the Ti 2p orbital resembles the trend observed in the O 1s orbital. The share of peaks in Ti 2p and O 1s clarifies that annealing under ambient air effectively oxidizes the TiO_*x*_ layer, accounting for a reduction in Ti^2+^ (TiO_*x*_) and an increment in Ti^4+^ species (TiO_2_) with respect to the as-deposited film. Another reason for the peak reduction at 531.6 eV (O 1s) is the dissociation of the C–O bond, which coincides with our observation in C 1s, where the percentage of the C–O peak area decreases after annealing. The reduction of the third peak in the O 1s spectrum indicates water desorption from the surface caused by annealing, and the remaining signal is mainly related to the SiO_*x*_ layer at the interface. In Si 2p core-level spectra of these samples, the peaks appeared at 98.6, 99.2, 100.15, 101.15, 102, and 102.85 eV are associated with Si 2p_3/2_, Si 2p_1/2_, Si_2_O (Si^1+^), SiO (Si^2+^), Si_2_O_3_ (Si^3+^), and SiO_2_ (Si^4+^), respectively.^9,24,56^ Inspecting the peak area percentage of silicon suboxides reveals that the nature of SiO_*x*_ differs in the studied samples. In RCA2-pretreated samples, annealing adds new suboxides rather than changing the overall oxide percentage. Nevertheless, comparing RCA2 with HF-dipped and UV–O_3_ samples, it is observed that the overall oxide percentage increases in the last two cases. Furthermore, the composition of UV–O_3_ oxide mostly consists of oxygen-rich (Si^3+^ and Si^4+^) species, whereas it does not hold for RCA2 oxide. These results agree with the observations in ref (57), where the UV–O_3_ method provides a more oxygen-rich oxide layer compared with wet chemically HNO_3_ and DIO_3_-grown oxides. Moreover, it has been demonstrated that among the wet-chemical oxidation methods, RCA2-grown oxides embrace the lowest oxygen density, or in other words, the highest density of oxygen vacancies (Ov).^58,59^ Although it is accepted that oxygen-rich oxide possesses fewer defects and provides better chemical passivation,^56,57^ Li et al. exhibited that silicon-rich oxide (i.e., the presence of Ov in the oxide layer) admits higher positive fixed charges (*Q*_f_) and induces more effective field-effect passivation on n-type silicon.^60^ Hence, the higher Ov density at the SiO_*x*_/TiO_*x*_ interface might explain the superior level of surface passivation achieved with RCA2-pretreated samples compared with those of HF and UV–O_3_ ones. The C 1s spectrum contains peaks corresponding to C–Si (283.5 eV), C–C (284.7 eV), C–O (286.6 eV), and C=O (288.5 eV). Applying annealing provides sufficient thermal energy to separate C from O, resulting in a reduced C–O percentage after annealing. Since the HF-dipped sample encounters higher and prolonged annealing temperature and duration (see Table S2), the decrease in C–O percentage is pronounced in the HF-dipped sample.

**Figure 4:**
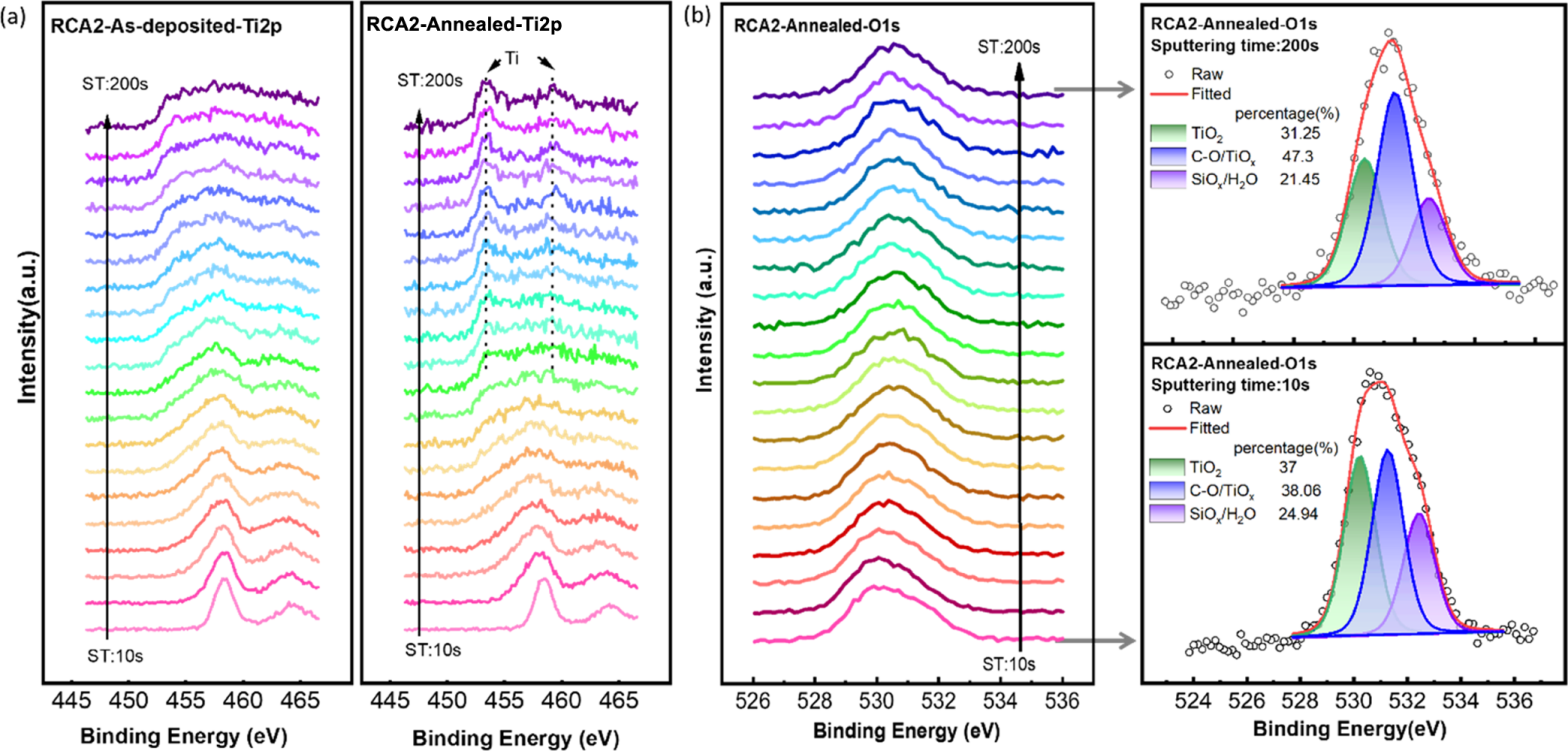
XPS depth profiling of the TiO_*x*_ film coated on the RCA2-pretreated substrates: (a) Ti 2p spectra for as-deposited and annealed (hot plate at 150 °C, 1 min) samples and (b) O 1s spectra of the annealed sample and its deconvolution for the spectra obtained after 10 and 200 s of sputtering etching time (ST denotes the sputtering etching time in seconds).

In addition, the chemical properties of the TiO_*x*_ film coated on the RCA2-pretreated substrate were evaluated by XPS depth profiling before and after annealing. The sputter etching time-dependent Ti 2p spectra shown in Figure 4a disclose that near the surface, the composition of TiO_*x*_ is more stoichiometric, but by approaching the Si/TiO_*x*_ interface, the spectrum widens and the proportion of oxygen-deficient species (i.e., Ti^2+^ and Ti^3+^) gradually increases. An analogous conclusion can be drawn from the O 1s spectrum of the annealed sample shown in Figure 4b; it indicates that as we get closer to the interface, the percentage of TiO_2_ and TiO_*x*_ peaks decreases and increases, respectively. Looking at the depth profile spectra of C 1s given in Figure S6c, the absence of the C–O peak (located at 286.6 eV) near the interface implies that the change in the share of the C–O/TiO_*x*_ peak in Figure 4b is solely caused by the increase in TiO_*x*_ content. Another piece of evidence for the existence of Ti lower oxidation states at the interface of the annealed sample is provided by the emergence of new peaks in the Ti 2p spectra. In ST:200 s, two peaks appear at 453.3 and 459.3 eV, which corresponds to the Ti metal or TiSi_2_; however, these peaks are not discernible in the as-deposited sample. We surmise that annealing fractures C–O–Ti bonds, causing released O and Ti to diffuse to the interface, thereby modulating their chemical and electrical properties. Diffusion of Ti and O toward interface upon annealing has been reported in other works as well.^11,32,61−63^ The surface and depth profiling XPS results suggest that annealing superficially oxidizes TiO_*x*_, reducing the Ov concentration close to the surface region, whereas, in the vicinity of the Si/SiO_*x*_/TiO_*x*_ interface, it leads to the appearance of Ti lower oxidation states and an augmentation in the Ov content. Consequently, annealing promotes the formation of a mixed oxide layer at the interface composed of substoichiometric SiO_*x*_ and TiO_*x*_, while it causes near-stoichiometric TiO_2_ composition at the surface.

**Figure 5:**
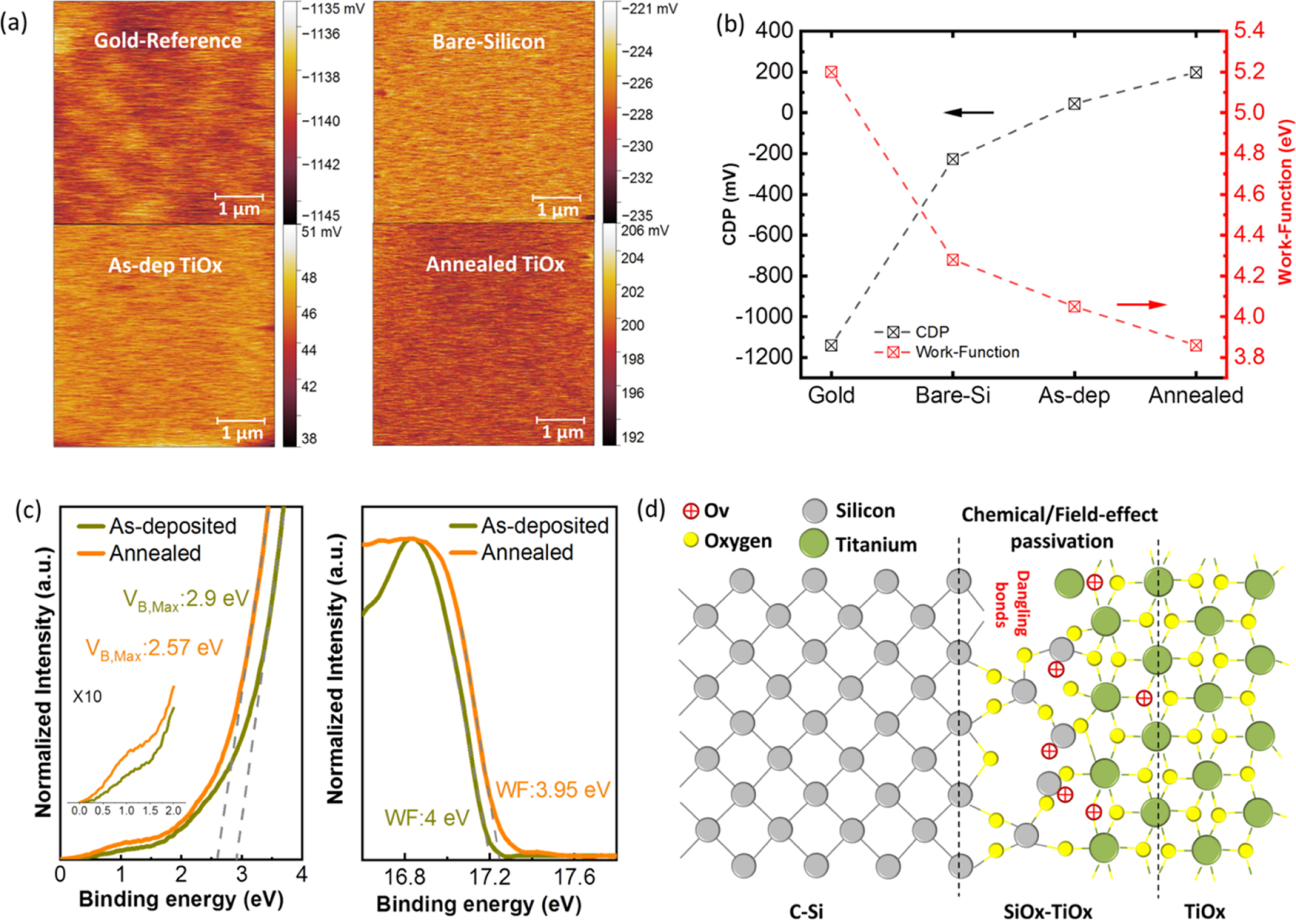
(a) CPD map, (b) CPD mean value and extracted WF of gold, bare, and TiO_*x*_-coated silicon samples measured by the KPFM technique, (c) UPS valence band (left) and secondary electron cutoff (right) spectrum of as-deposited and annealed TiO_*x*_, (d) schematic illustration of chemical and electrical properties of the Si/SiO_*x*_/TiO_*x*_ interface. Herein, to visually represent the electrical influence of lower oxidation states of Ti and Ov in the mixed oxide layer, the Ov content is symbolized as positive fixed charges. (Annealing of the TiO_*x*_ sample was performed at 150 °C for 1 min on a hot plate in the ambient room; the bare Si sample received RCA2 treatment before CPD measurement).

Capacitance–voltage (*C*–*V*) and Kelvin probe force microscopy (KPFM) were conducted to inspect the passivation mechanism in more detail. The hysteresis *C*–*V* curves of as-deposited and annealed samples measured at 1 MHz are shown in Figure S7. There are two distinct and prominent effects present on the *C*–*V* behavior of the sample upon annealing: First, although the as-deposited sample exhibits a clear hysteresis characteristic, no such vivid behavior is seen after annealing. Hysteresis is due to the slow states or defect sites mainly responsible for the trapping of charges.^64−66^ These slow states are often referred to as border traps^67^ to distinguish it from the conventional connotation of interface states. The interfacial unpassivated slow defect sites get positively charged during the forward scan, causing an apparent negative flat-band (*V*_fb_) shift in the reverse bias in the as-deposited sample. Second, there is a reduced stretch-out of the high-frequency capacitance along the voltage axis after annealing. The occurrence of stretch-out is attributed to the presence of interface traps.^68−70^ The elimination of hysteresis and the sharp transition from the inversion to accumulation regions (and vice versa) after annealing can therefore be ascribed to the suppression of slow defect sites and interface trap density brought about by promoted chemical passivation. While negative *V*_fb_ and positive *Q*_f_ of −0.44 V and 4.84 × 10^11^ cm^–2^ were calculated for the annealed sample, respectively, the high current leakage across the device precluded the extraction of *D*_it_ via conductance-based methods to quantify the chemical passivation level. There have been reports about the potential presence of both positive and negative *Q*_f_ in the TiO_*x*_ layer, which in fact depends on the chemicals employed in the fabrication process, the Ti precursor, the deposition technique and conditions, as well as pre- and postdeposition treatments.^12,71,72^ Our observations concerning TDIP (the titanium precursor used in this study) ability to generate substoichiometric TiO_*x*_ and a negative V_fb_ as the evidence of positive *Q*_f_ are aligned with the results reported in ref (38). The KPFM technique is used to measure the contact potential difference (CPD) between the tip and the surface of the sample. The type of charges and energy band alignment at the interface governs the amplitude and polarity of CPD. The CPD map and its corresponding mean values for various samples are shown in Figure 5a,5b, respectively. The CPD mean values were obtained from the voltage distribution profile plotted in Figure S8. Given that the KPFM setup was not calibrated in our experiments, a gold-coated sample is used as a reference to extract the WF (through eq 3 written in the Materials and Methods section). Assuming 5.2 eV as the WF of the reference sample, WFs of 4.28, 4.0, and 3.85 eV were calculated for bare Si, as-deposited TiO_*x*_, and annealed TiO_*x*_, respectively, as depicted in Figure 5b. The WF reduction of TiO_*x*_ is evident upon annealing. A similar tendency was ascertained by the ultraviolet photoelectron spectroscopy (UPS) secondary electron cutoff spectrum shown in Figure 5c (right). It revealed that the WF of TiO_*x*_ decreases from 4.0 to 3.95 eV after annealing. It is worth mentioning that the UPS measurement gathers the electrons excited from the first 1 nm of the surface, which is composed of oxidized TiO_*x*_. Therefore, it is likely to observe a more pronounced WF difference between as-deposited and annealed TiO_*x*_ by approaching the Si/TiO_*x*_ interface, where Ov and lower Ti oxidation states are dominant in the annealed sample. Based on the results, it can be inferred that the lower WF of TiO_*x*_ with respect to Si induces a dipole directed from TiO_*x*_ to Si, accumulating electrons and increasing the e/h ratio at the interface. This conclusion can also be drawn by comparing the CPD values of the bare silicon and TiO_*x*_-coated samples. After annealing, there is a significant discrepancy of 430 mV in CPD between the bare Si and annealed samples, implying an intense downward band bending and electron accumulation region at the Si/SiO_*x*_/TiO_*x*_ interface.^73−75^ The variation in CPD and WF upon the annealing could be explained via the fact that Ov in the TiO_*x*_ structure acts like an n-dopant by leaving two electrons behind (in Ti-3d orbital), altering its electronic nature from insulator to semiconductor.^63,76^ In addition, by magnifying the valence band spectrum shown in Figure 5c (left), it becomes evident that annealing generates new states around 1 eV associated with the lower Ti oxidation states,^76^ aligning with the findings from the depth profiling XPS.

Eventually, based on the diverse characterization results, it can be interpreted that the improved electrical properties seen in the tailor-made Si/SiO_*x*_/TiO_*x*_ interface under the optimal processing condition are attributed to a synergistic interplay between chemical and field-effect mechanisms, as schematically illustrated in Figure 5d. Nevertheless, the inferior passivation quality obtained on p-type c-Si (Figure S9) acknowledges the eminent contribution of field-effect passivation. Liao et al. demonstrated that when the surface passivation is dictated by chemical passivation, almost identical τ_eff_ is observed on n- and p-type silicon.^12^ The attained low contact resistivity is also owing to both chemical and field-effect mechanisms, which can be construed as follows: (i) Chemical passivation screens the dangling bonds and trap states at the interface hindering Fermi-level pinning, even in the absence of LiF_*x*_ interlayer. (ii) It is well-known that TiO_*x*_ with a higher concentration of Ov yields higher conductivity and thus lower ρ_c_.^6,19,32,35^ The overlaying metal can affect the TiO_*x*_ composition, which indeed depends on the metal work function.^15,77^ The redox reaction of TiO_*x*_ enhances when it comes in contact with a low WF overlayer, leading to a reduction in ρ_c_.^8,78,79^ (iii) Meanwhile, as probed by AFM (Figure S4b), longer annealing time coincides with the development of valleys or pinholes, which, first, promotes current flow through defect-assisted tunneling and, second, facilitates LiF_*x*_ diffusion in the TiO_*x*_ underlay, evoking energy states close to the conduction and/or valence bands of TiO_*x*_ and thus exciting its conductivity.^43^ Because of the aforementioned reasons, we observed a lower ρ_c_ as LiF_*x*_ and/or a longer annealing duration was employed (see Figure 2c). Our detailed experimental study enlightens the fact that the presence of Ov and Ti lower oxidation states at the interface are the main factors responsible for the enhanced electrical properties of the optimized TiO_*x*_ in this work and the engineering of these factors’ content at the Si/TiO_*x*_ interface could be adopted for further improvement.

## 4 Conclusions

In conclusion, we have demonstrated an effective route to raise the solution-processed TiO_*x*_ electrical properties to a level comparable with that of the state-of-the-art TiO_*x*_-based CSCs deposited under vacuum. Our findings demonstrate that both pre- and postdeposition treatments play a pivotal role in the electrical properties of Si/SiO_*x*_/TiO_*x*_ heterojunction. Excellent surface passivation prior to metallization (i*V*_oc_: 706 mV) and low contact resistivity (ρ_c_: 15.4 mΩ·cm^2^) are simultaneously achieved through RCA2 pretreatment and a brief low-temperature post-treatment on the hot plate in the ambient room. Experimental characterizations elucidate that applying the optimal annealing step not only activates the chemical passivation by forming Ti–O–Si and Si–O–Si bonds at the interface but also induces the field-effect passivation and electron selectivity features by modulating Ov and Ti states, leading to the formation of positive fixed charges at the interface. This study highlights a way to fabricate high-quality solution-processed TiO_*x*_-based electron-selective passivation contacts via a simple and low-temperature fabrication procedure, introducing a promising strategy for high-performance-to-cost ratio c-Si heterojunction solar cells.

## Supporting Information Available

Thickness and optical properties extraction by spectroscopic ellipsometry (SE); verification of passivation parameters reproducibility; dark current–voltage (*I*–*V*) curves of TLM structures, atomic force microscopy images; attenuated total reflectance Fourier transform infrared (ATR-FTIR) and transmission FTIR spectrum, XPS general survey and quantitative analysis of Ti 2p, O 1s, Si 2p, and C 1s core-level spectra, capacitance–voltage (*C*–*V*), contact potential difference (CPD) distribution profiles, and photoluminescence images (PLI) of TiO*_x_* coated on n- and p-type c-Si wafers (PDF)
