# Supplementary material for: Optimization of a Solution-Processed TiOx/(n)c-Si Electron-Selective Interface by Pre- and Postdeposition Treatments
Source: ACS Appl Mater Interfaces. 2024 Mar 19;16(13):16950–61. doi: 10.1021/acsami.3c18134 (PMC10995901; doi:10.1021/acsami.3c18134)
Supplement: Supplementary file 1 — am3c18134_si_001.pdf [file am3c18134_si_001.pdf]

Supporting information for:

Optimization of solution-processed TiO_x/(n)c-Si electron selective interface by Pre- and Post-Deposition Treatments

*Naser Beyraghi [a,b], Mehmet C. Sahiner [a,c], Oguzhan Oguz [a], Selcuk Yerci [a,b,c]**

Email: syerci@metu.edu.tr

[a] ODTU-GUNAM, Middle East Technical University, Ankara 06800, Turkey

[b] Department of Micro and Nanotechnology, Middle East Technical University, Ankara 06800, Turkey

[c] Department of Electrical and Electronics Engineering, Middle East Technical University, Ankara 06800, Turkey

The optical constants (refractive index, n, and k), and thickness of annealed TiO_x film coated on RCA2-pre-treated silicon substrates were determined by spectroscopic ellipsometry measurements. The recorded Cos (Delta) and Tan (Psi) spectra were fitted with the Tauc-Lorentz dispersion model to extract n, k, and thickness, as shown in **Figure S1**. The thickness of the TiO_x film was studied as a function of the dilution ratio. The reported thickness (7.1 nm) for a dilution ratio of 1:3 is the average of ten separate points.

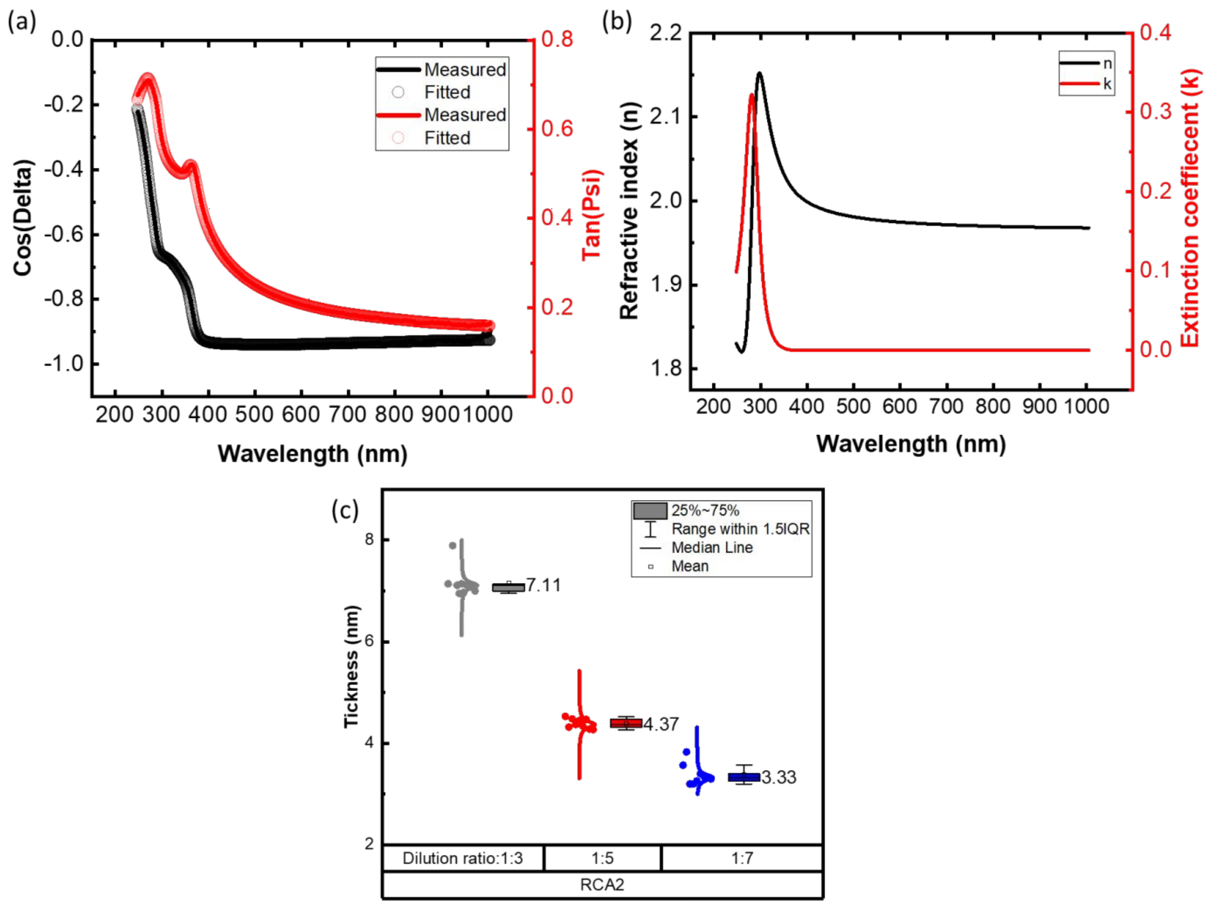

Figure S1. (a) Cos(delta) and Tan (Psi) spectra measured by spectroscopic ellipsometry (SE) and fitted by the Tauc-Lorentz dispersion model; (b) Variation of refractive index (n) and extinction coefficient (k) versus photon energy; (c) The thickness dependence of spin-coated film on the solution dilution ratio, averaged from ten separate points. (All measured samples are RCA2 pre-treated and annealed at 150 ˚C for 1min after TiO_x deposition)

To establish the reproducibility of the passivation produced by TiO_x film coated on the RCA2 pre-treated substrate and post-annealed on the hotplate, at least three passivation samples were

prepared for each orange-colored data point presented in **Figure 1c**. **Figures S2a** and **b** demonstrate an observable statistical trend in the measured iV_{oc} and effective lifetime versus annealing temperature and duration, emphasizing that annealing at 150 ˚C for 1 min is the optimal annealing condition. **Figures S2c** illustrates the injection-level-dependent effective lifetime of the samples annealed at optimal hotplate temperature of 150 ˚C for different annealing durations. In terms of stability, we found that the passivation quality of the investigated TiO_x strictly depends on the storing condition. As depicted in **Figure S2d**, the iV_{oc} of a fresh sample produced under optimal condition drastically degrades over time, as the sample is kept in the room ambience, reaching 555 mV after nine days. On the contrary, iVoc remains almost unchanged when the sample is kept in the nitrogen box during the same period of time. This experiment was performed to figure out how the passivation quality can be preserved before cell fabrication. One possible explanation for passivation degradation is the gradual annihilation of oxygen vacancies caused by the interaction with the hydroxyl and oxygen radicals present in the ambient room. In fact, N_2 ambient mimics encapsulated (oxygen- and water-free region) Si/TiO_x stack, and effectively retains its passivation quality.

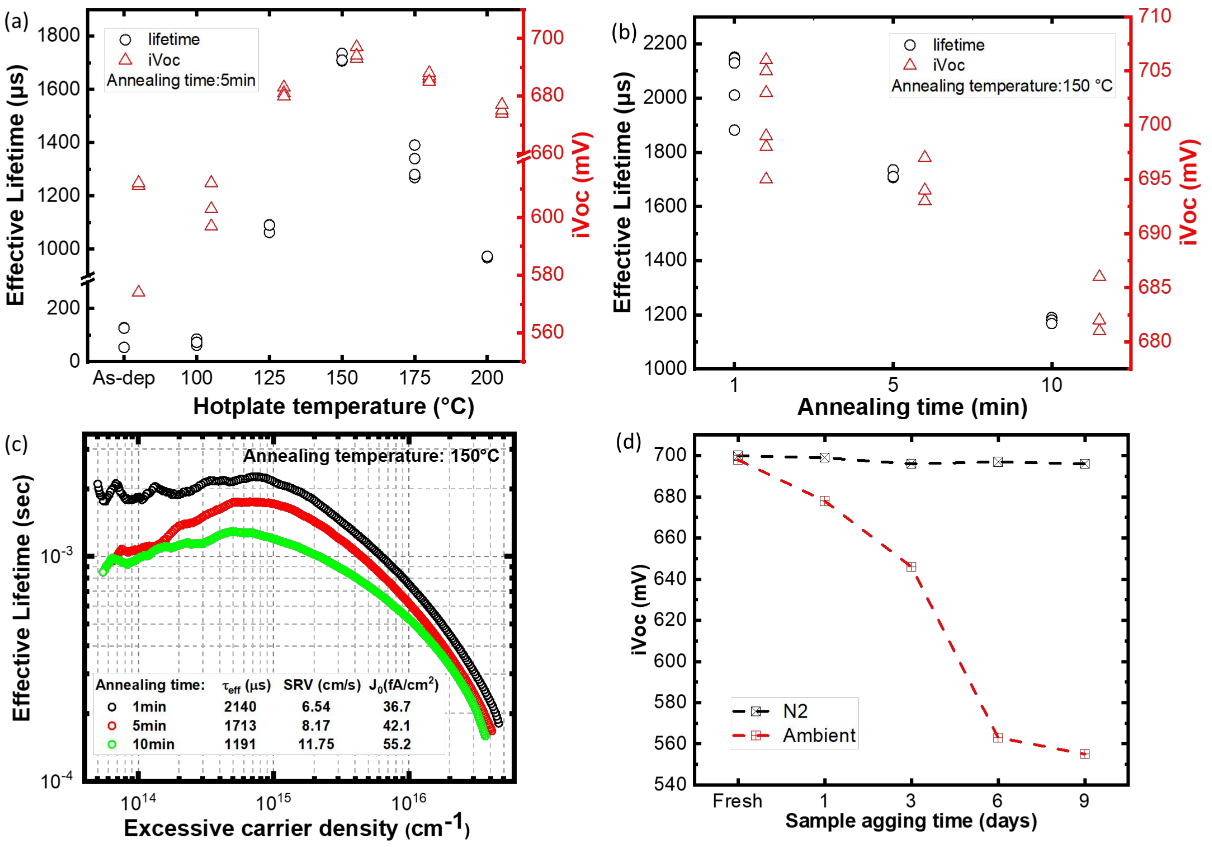

Figure S2. Reproducibility of the passivation quality produced at (a) fixed 5min for different annealing temperatures, and (b) fixed 150 ˚C for different annealing durations, (c) Injection-level-dependent effective lifetime of the samples annealed at optimal hotplate temperature of 150 ˚C for different annealing durations (legend presents the extracted τ_{eff}, SRV and J_0 at the excessive carrier density of $1x10^{15}cm^{-1}$), (d) time-dependent stability of passivation samples in room and N_2 ambiences. (All samples received RCA2 treatment before TiO_x coating and annealing was carried out on the hotplate). The dashed lines on (d) are provided to guide the eye.

Transmission Line Method (TLM) was utilized to extract the contact resistivity (ρc) of n-Si/TiO_x/Al and n-Si/TiO_x/LiF_x/Al hetero-junctions. As shown in **Figure S3a**, both sides of the TLM test structures were laser-cut prior to the dark current-voltage (I-V) measurement to confine the lateral spreading contribution. The dark I-V curves of samples with Al and LiF_x/Al contact types were presented in **Figures S3b** and **3c**, respectively. The sample in direct contact with Al, exhibits the lowest drawn current and non-linear behavior within the applied voltage range, implying a relatively high Schottky barrier height at the interface caused by metal-induced-gap states (MIGS) and Fermi-level pinning that obstructs electron transportation and prevents ρc extraction. By incorporating TiO_x and, in particular, TiO_x/LiF_x interlayers, the drawn current increases and exhibits linear ohmic behavior, allowing ρc extraction. This signifies that the use of TiO_x and TiO_x/LiF_x interlayers can effectively screen the MIGS and reduce the defect density at the interface, resulting in Fermi-level un-pinning and barrier height reduction.

(a)

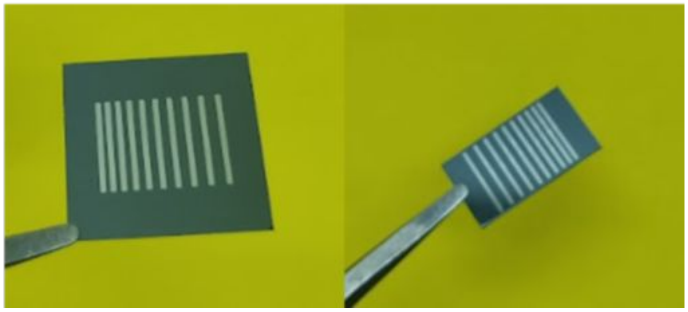

(b)

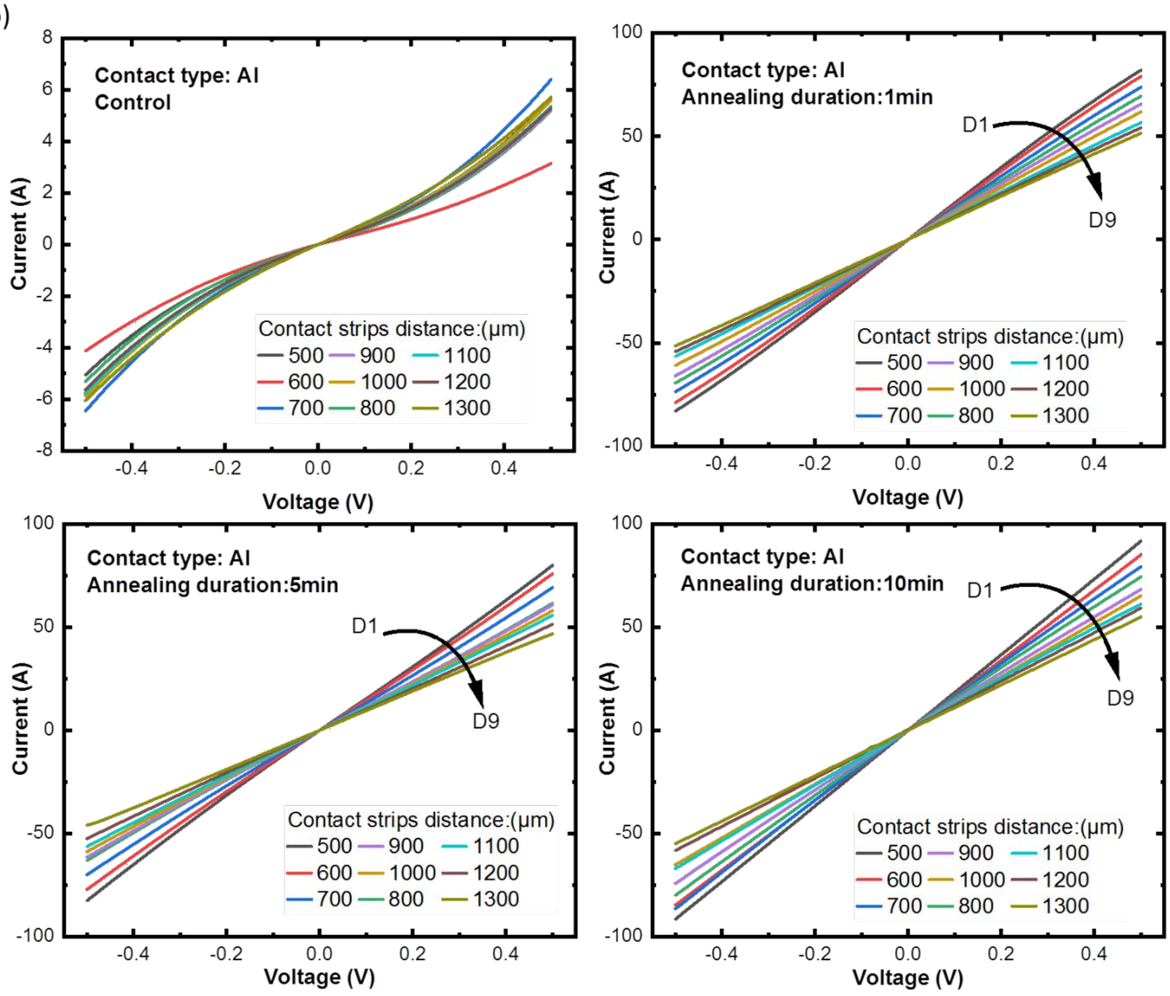
Contact type: Al
Control
Current (A)
Voltage (V)
Contact strips distance:(μm)
500 900 1100
600 1000 1200
700 800 1300
Contact type: Al
Annealing duration:1min
D1
D9
Current (A)
Voltage (V)
Contact strips distance:(μm)
500 900 1100
600 1000 1200
700 800 1300
Contact type: Al
Annealing duration:5min
D1
D9
Current (A)
Voltage (V)
Contact strips distance:(μm)
500 900 1100
600 1000 1200
700 800 1300
Contact type: Al
Annealing duration:10min
D1
D9
Current (A)
Voltage (V)
Contact strips distance:(μm)
500 900 1100
600 1000 1200
700 800 1300

(c)

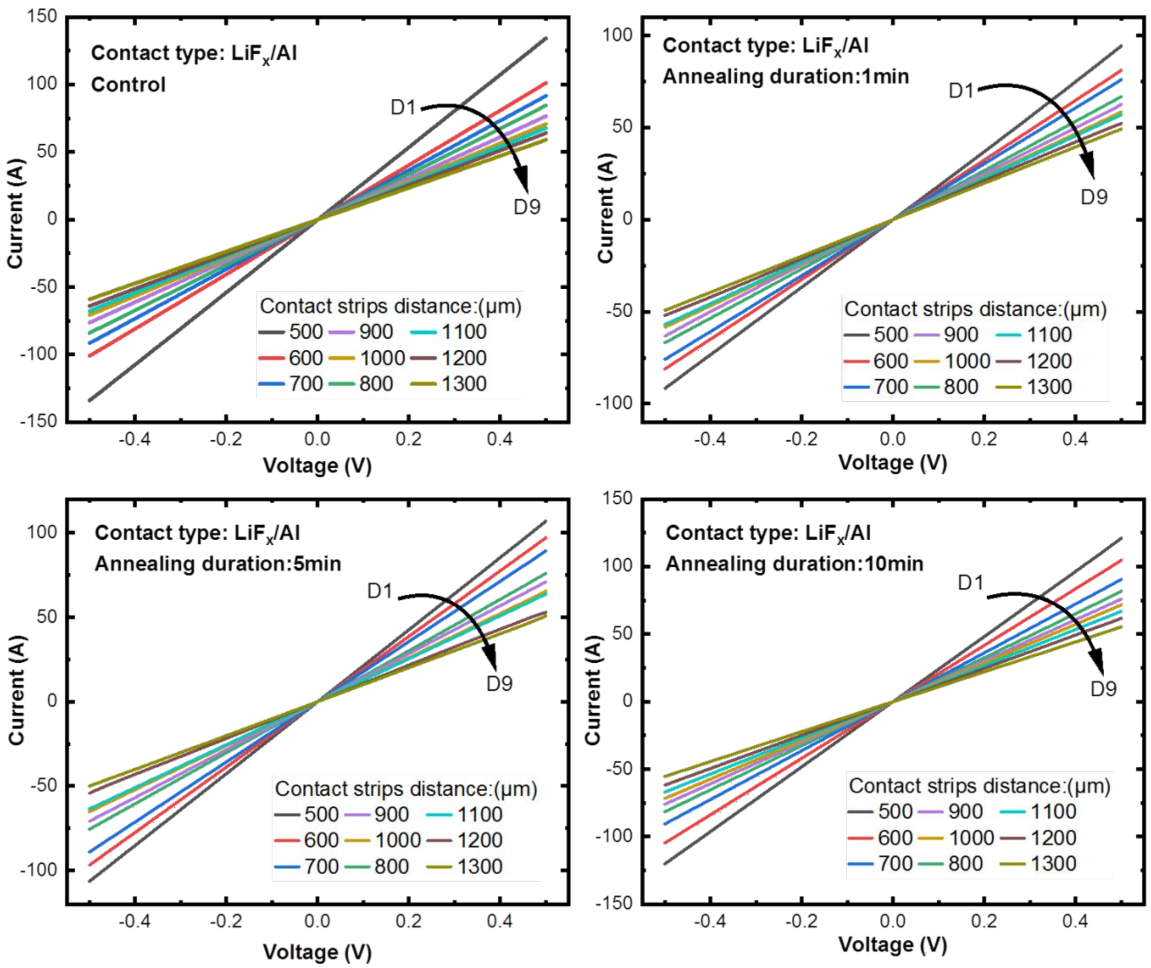

Figure S3. (a) The edge-cut of TLM test structure to confine the lateral spreading contribution; before laser-cut (right), after laser-cut (left); dark I–V characteristics of (b) n-Si/TiO_x/Al and, (c) n-Si/TiO_x/LiF_x/Al structures annealed at 150 ˚C for 1, 5, and 10 min after TiO_x deposition on RCA2 pre-treated substrates.

Atomic Force Microscopy (AFM) is used to assess the temperature-dependent structural variation of the TiO_x layer spin-coated on an RCA2 pre-treated silicon substrate. After deposition, samples were subjected to annealing at 100, 150, and 200 ˚C for 1min and at 150 ˚C for 5- and 10-min. **Figure S4a** depicts the relevant surface morphology and the distribution of surface root mean square (RMS) roughness. It is evident that elevating the annealing temperature and extending the annealing duration roughen the surface. For the as-deposited sample, the average RMS roughness is 0.18 nm, while annealing at 100, 150, and 200 ˚C for 1 min increases it to 0.33, 0.27, and 0.48 nm, respectively. Moreover, longer annealing durations (5 and 10 min) at 150 ˚C harshly roughen the surface, resulting in a substantial increase in RMS roughness (1.15 and 1.35 nm). **Figure S4b** displays the corresponding 2D-AFM images of these samples along with a line profile, revealing that longer annealing durations (5 and 10 min) create deep valleys or even pinholes in the TiO_x film. For instance, an approximate peak-to-valley height of 6 nm is measured after 10 min of annealing at 150 ˚C, indicating the formation of pinholes on this sample. Among the annealed samples, the lowest roughness belongs to the sample annealed at 150 ˚C for 1 min, which yielded the highest passivation quality as well. In addition, referring to **Figures 1c** and **1e**, we observe that

the iV_{oc} decreases as samples are subjected to annealing at 200 ˚C and at 150 ˚C for 5 and 10 min. According to these results, we speculate that the passivation degradation at higher temperatures and longer durations depends somewhat on the surface roughening. However, the tendency in surface morphology changes is not entirely in line with that in iV_{oc}. For instance, the sample annealed at 100 ˚C has a smoother surface than that of the sample annealed at 200 ˚C; but, in terms of passivation the latter one has higher quality.

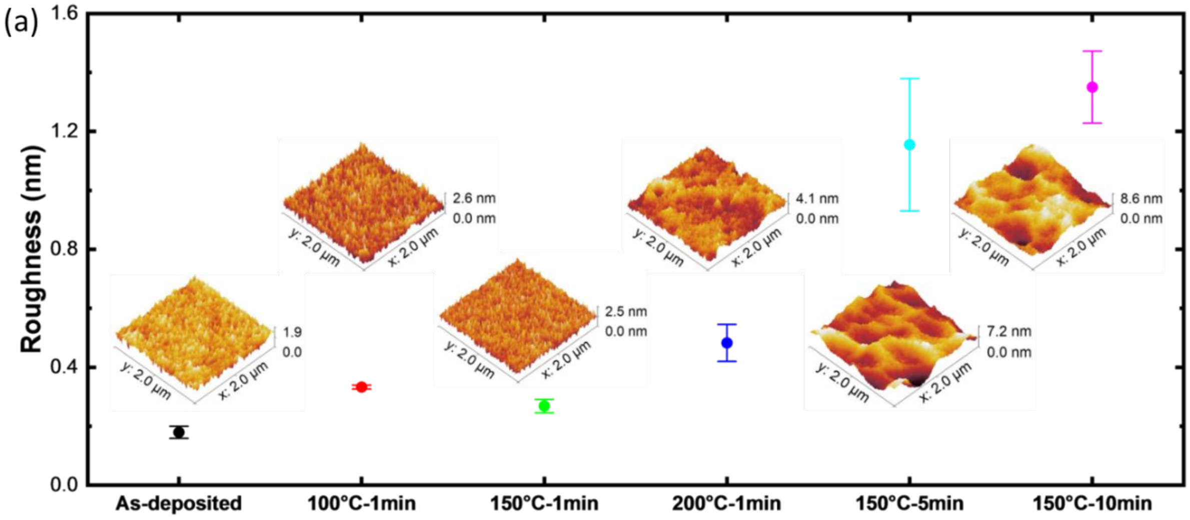

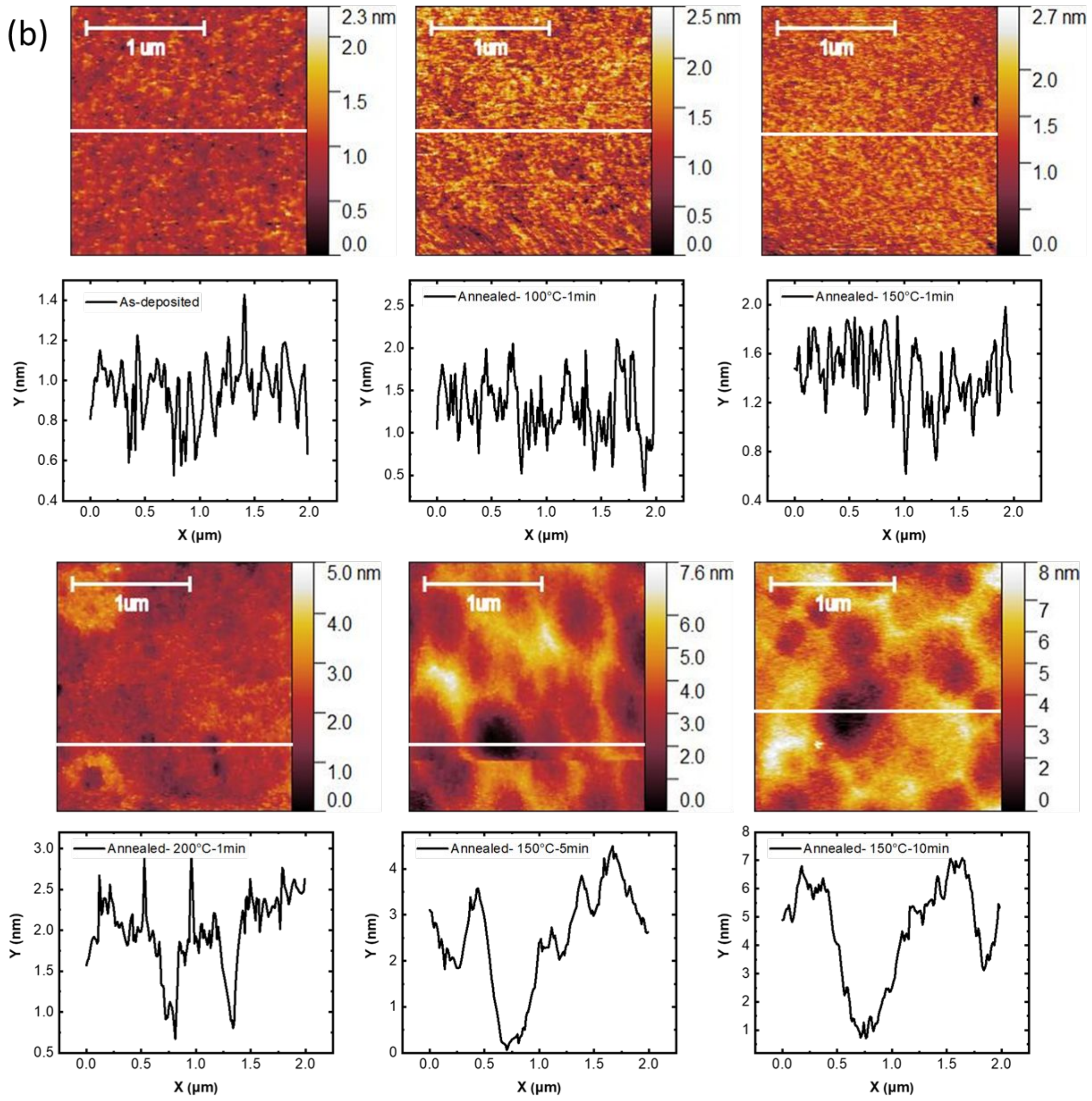

Figure S4. (a) 3D-AFM images and distribution of surface RMS roughness, (b) 2D-AFM images and line profile taken from the white line drawn on each image. (All samples received RCA2 treatment before TiO_x coating and annealing was carried out on the hotplate)

The TiO_x solution is a mixture of 1-butanol, TDIP, and DIW solvents. **Figure S5a** depicts the full absorption spectrum (650–4000 cm^{-1}) of individual solvents. The absorption spectrum of TiO_x solution is a replica of 1-butanol (not shown here), which constitutes almost 98% of its volume in 1:3 diluted solution. The prominent and distinct absorption peaks highlighted in the (II), (III), and (IV) regions represent vibrational modes of methyl and hydroxyl groups, respectively. Moreover, peaks detected within the region (I) are assigned to the C-O (-C) vibration modes. The detailed designations of the detected peaks are presented in **Table S1**. Absorption spectra of 1-butanol, TDIP, and TiO_x coated on the RCA2 pre-treated silicon substrate in the fingerprint region were shown in **Figure S5b**. On silicon, the characteristic vibrational modes pertaining to different Si, O, and Ti interactions have been defined, however, 850-1150 cm^{-1} range includes wavenumbers assigned to the carbon compounds superimposed with other characteristic peaks. For example, 1044 cm^{-1}, 1055 cm^{-1}, and 1067 cm^{-1}, were assigned to C-O stretching, and C-O-C stretching vibrations.[1, 2, 8-10] Consequently, due to the less porous layer and a higher amount of carbon-related compounds in the as-deposited state, the peaks are more prominent than that in the annealed sample.

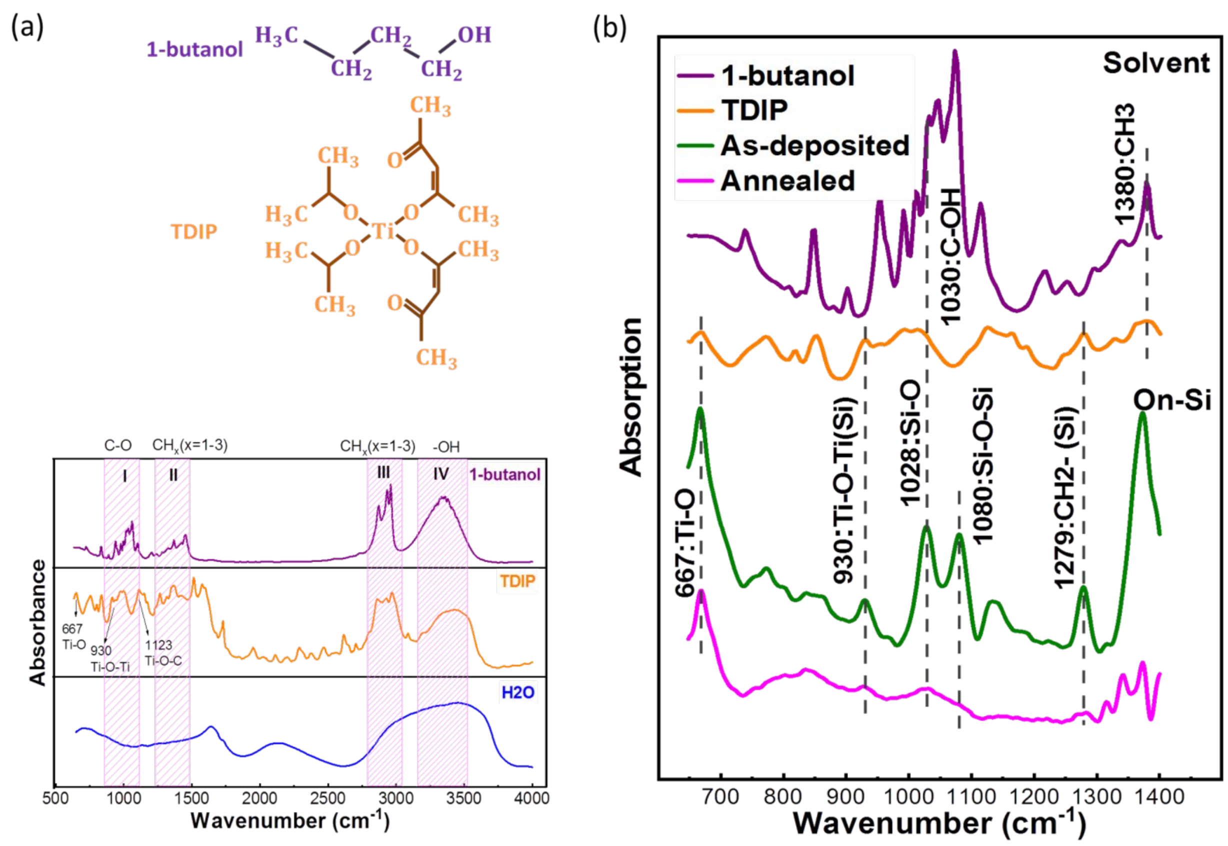

Figure S5. (a) Full absorption spectrum (650-4000 cm^{-1}) of the solvents constitute the TiO_x solution measured by Fourier transform infrared (ATR-FTIR); (b) Absorption spectra of 1-butanol, TDIP, and TiO_x coated on the RCA2 pre-treated silicon substrate in the fingerprint region (i.e., below 1400 cm^{-1}).

X-ray photoelectron spectroscopy (XPS) was used to study the chemical composition and oxidation state of the samples described in **Table S2**. In all samples, the survey scan identified peaks associated with C, O, Ti, and Si elemental species illustrated in **Figure S6a**. **Figure S6b** displays the peak area percentage of bindings detected in the C1s, O1s, Ti2p, and Si2p core level spectra (Note: These percentages refer to regions close to the TiO_x surface). **Figure S6c** compares the C1s orbital of as-deposited and annealed sample as a function of sputter etching time. It indicates that the C-related components abruptly decrease after only a few steps of sputtering in the annealed sample, while it can be detected for longer sputtering time in the as-deposited sample. This implies that the content of carbon in as-deposited sample is higher than the annealed one, supporting our notion about the fracture of C-O-Ti bond upon the annealing. Furthermore, there is a shift towards lower binding energies as sputtering time increases. This indicates that by approaching the interface carbon components of C-C (284.7 eV), C-O (286.6 eV), and C=O (288.5 eV) disappear and instead it appears in the form of C-Si (283.5 eV) and C-Ti (282 eV).[11, 12] Hence, it can be asserted that the change in the share of the C-O/TiO_x peak from the surface to interface

shown in **Figure 4b** is solely caused by the increase in TiO_x content, providing more evidence for the evolution of sub-stoichiometric TiO_x in the regions close to the Si/TiO_x interface.

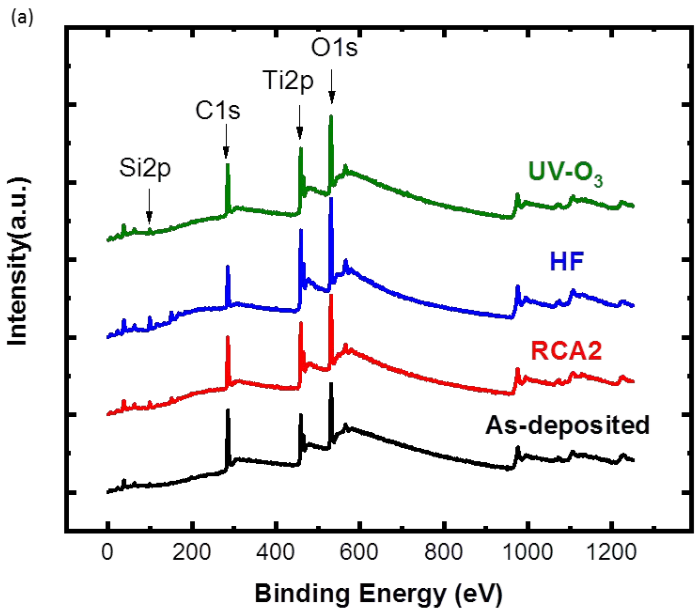

(b)

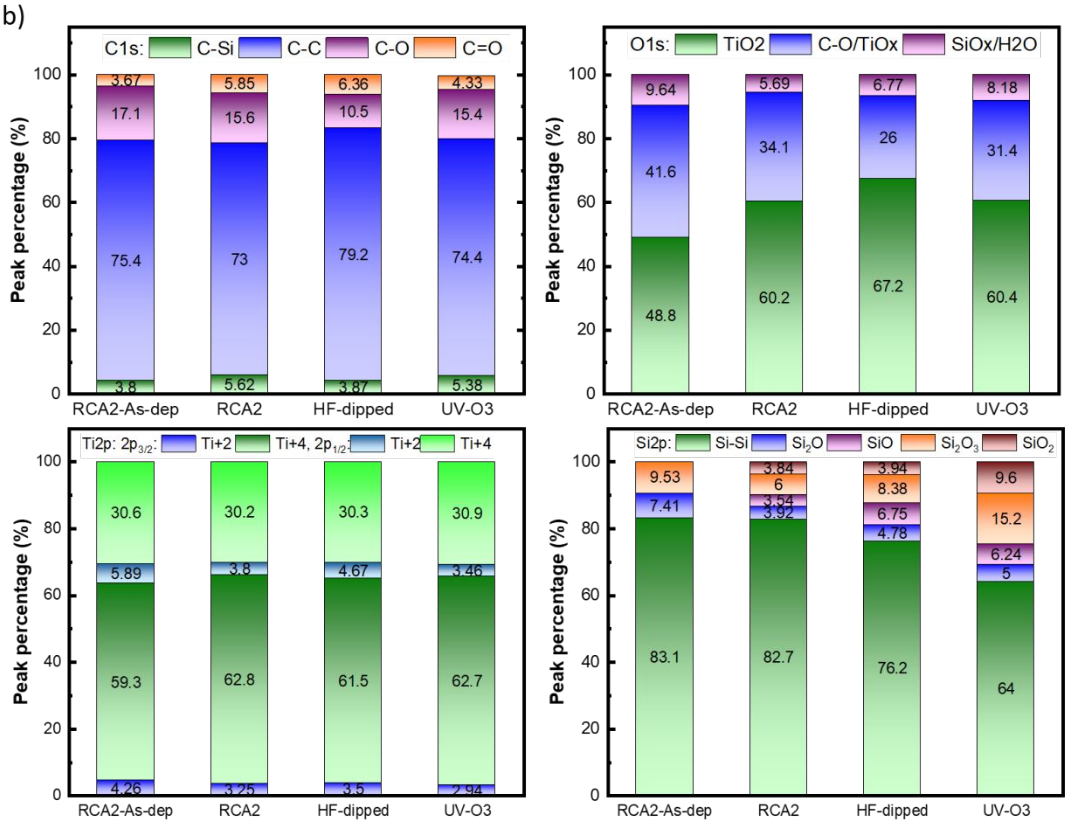
C1s: C-Si C-C C-O C=O
Peak percentage (%)
RCA2-As-dep
RCA2
HF-dipped
UV-O3
3.67
17.1
75.4
3.8
5.85
15.6
73
5.62
6.36
10.5
79.2
3.87
4.33
15.4
74.4
5.38
O1s: TiO2 C-O/TiOx SiOx/H2O
9.64
41.6
48.8
5.69
34.1
60.2
6.77
26
67.2
8.18
31.4
60.4
Ti2p: $2p_{3/2}$: Ti+2 Ti+4, $2p_{1/2}$: Ti+2 Ti+4
30.6
5.89
59.3
4.26
30.2
3.8
62.8
3.25
30.3
4.67
61.5
3.5
30.9
3.46
62.7
2.94
Si2p: Si-Si Si_2O SiO Si_2O_3 SiO_2
9.53
7.41
83.1
3.84
6
3.54
3.92
82.7
3.94
8.38
6.75
4.78
76.2
9.6
15.2
6.24
5
64

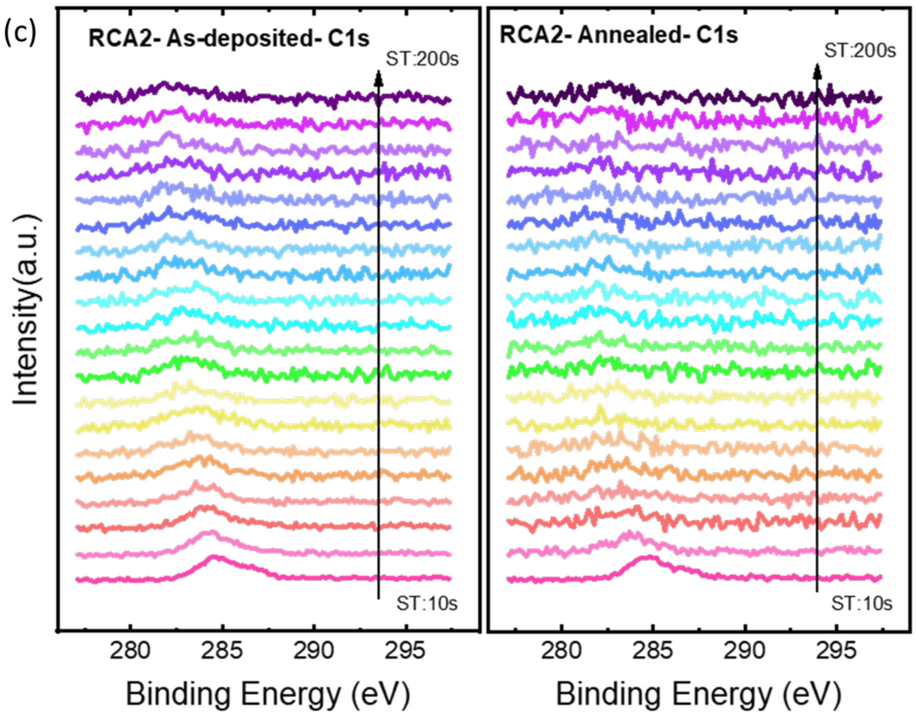

Figure S6. (a) Survey XPS scan of samples prepared under the condition described in **Table S2**; (b) the percentage of area under the deconvoluted peaks in C1s, O1s, Ti2p, and Si2p core level spectra, (c) comparison of the depth profile C1s spectrum for as-deposited and annealed sample coated on RCA2 pre-treated sample.

The CV test samples were fabricated on RCA2 pre-treated silicon substrates. A bilayer of LiF_x/Al (1/200 nm) fully evaporated on the rear side to form an ohmic contact to eliminate capacitance contribution from the back contact. On the front side Al contact evaporated on Spin-coated TiO_x through a shadow mask patterned with circle openings with a radius of 350 μm. The

inset in **Figure S7** schematically describes the fabricated structure. The normalized capacitance-voltage curves obtained via cyclic sweeping of bias from inversion into the accumulation and back to inversion. Both the hysteresis and the stretch-out behavior of capacitance on bias of as-deposited sample are significantly reduced after annealing. The measured capacitance was normalized with respect to the oxide capacitance empirically determined via method introduced in Reference (13). The apparent reduction in accumulation capacitance beyond a certain voltage stems from the series resistance of the sample, which exhibits a significant bias-dependence coupled with current leakage, is no simple task to correct without extensive impedance modeling. Nevertheless, its parasitic effect is negligible around the locus of interest, where hysteresis is prominent: starting from inversion, going over depletion up to the onset of accumulation.

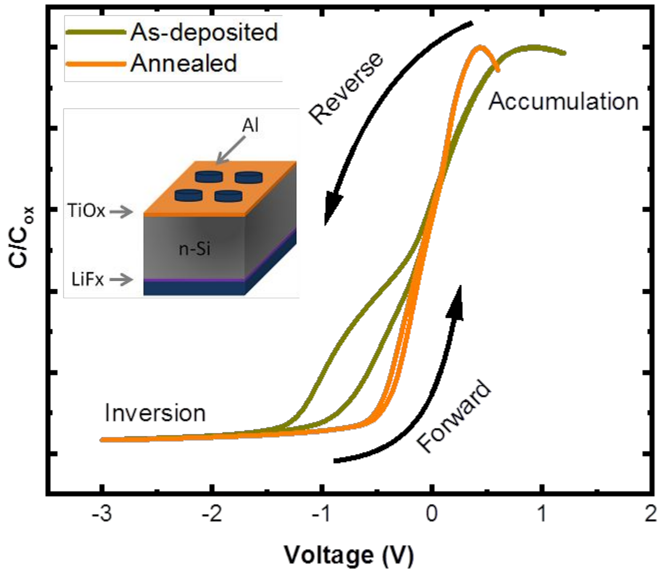

Figure S7. Forward and reverse C–V characteristics of the as-deposited and annealed samples in the range of -3 to +1.5 V applied voltage at 1 MHz. (inset shows the CV test sample structure)

The voltage distribution of the CPD maps presented in **Figure 5a** was plotted to find its mean value, as shown in **Figure S8**. The CPD mean value is utilized for calculation of work function reported in **Figure 5b**.

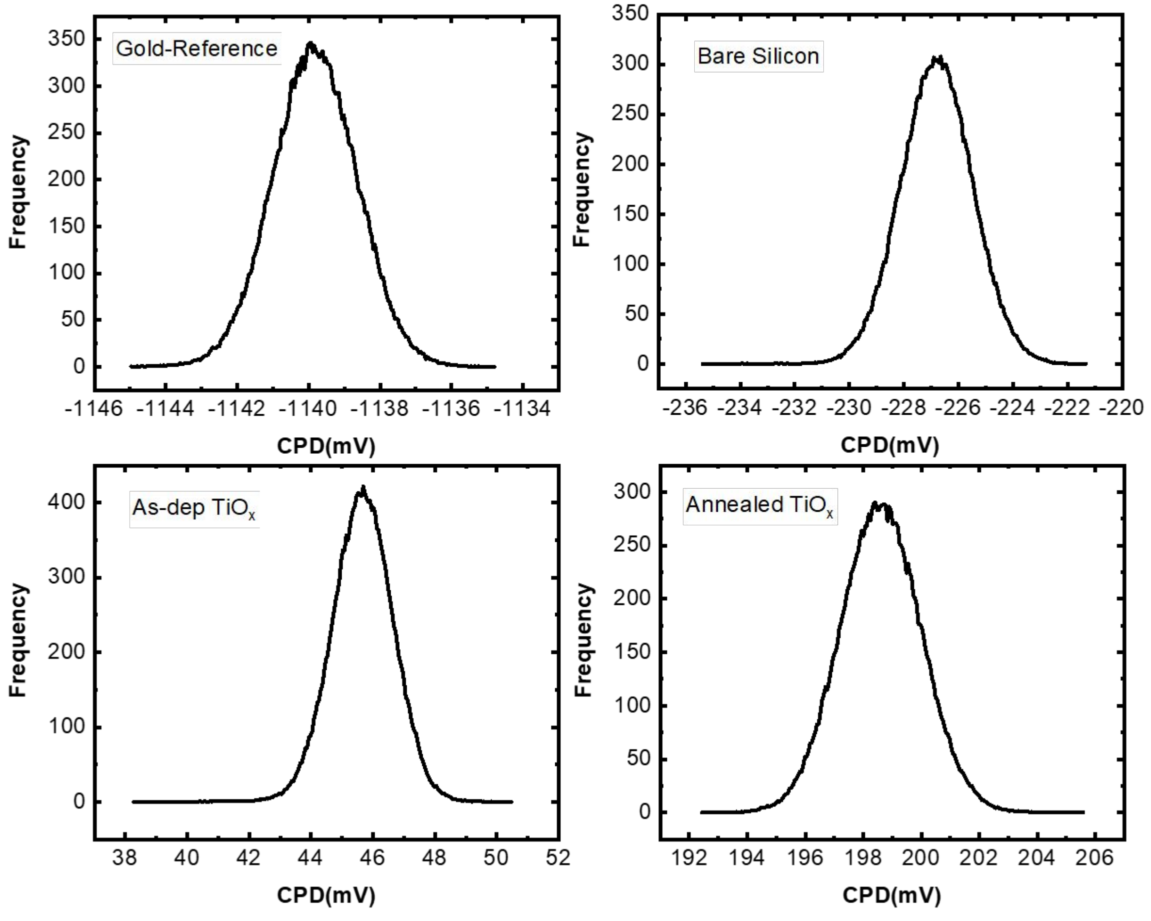

Figure S8. The corresponding voltage distribution profile of the CPD maps presented in **Figure 5a**.

We believe that the outstanding level of surface passivation obtained on n-type silicon is due to a combination effect of chemical and field-effect passivation features in the investigated $Si/SiO_x/TiO_x$ hetero-junction. Nonetheless, the inadequate surface passivation gained on p-type silicon emphasizes the significant influence of field-effect passivation. Considering the fact that the built-in potential induced by TiO_x film directs from TiO_x to silicon (i.e., a downward band bending at the $Si/SiO_x/TiO_x$ interface), it can be inferred that holes, the majority carriers in the p-type silicon, will be repelled from the interface while electrons, the minority carriers, will be attracted to it. Thus, the concentration of minority carriers increases at the interface, which favors the recombination mechanism. **Figure S9** compares the passivation quality on n- and p-type silicon substrates. To keep the bulk quality identical in n- and p-type silicon substrates, for both cases CZ wafer with thickness of 280 μm and resistivity of 1-3 Ω.cm were used. As seen in **Figure S9a**, the iV_{oc} enhances after annealing at 150 °C for 1 min on both silicon types as evidence of chemical passivation activation, but its enhancement on p-type is negligible with respect to n-type silicon and halts at approximately 610 mV. The discrepancy in passivation levels on n- and p-type silicon is also qualitatively clarified by photoluminescence (PL) images, as shown in **Figure S9b**.

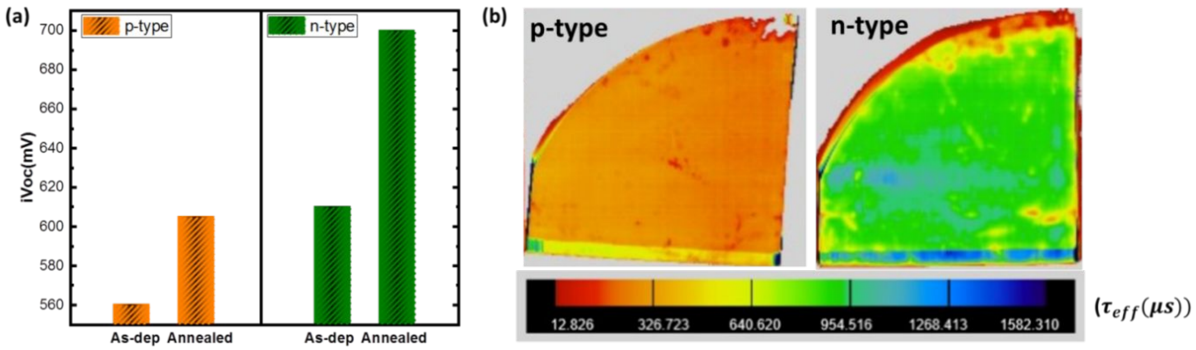

Figure S9. Passivation level on n and p-type silicon substrates characterized by (a) quasi-steady state photoconductance (QSSPC), and (b) photoluminescence (PL) imaging techniques.

Table S1. Assignment of peaks detected from ATR spectral data for individual solvents.

TDIP			1-butano		
Group	Wavenumber (cm^{-1})	Ref	Group	Wavenumber (cm^{-1})	Ref
OH group stretching	3100-3500	[1, 2]	OH group stretching	3100-3500	[1, 2]
C-H asymmetric stretching in CH3	2971	[1, 2]	C-H asymmetric stretching in CH3	2963	[1, 2]
C-H asymmetric stretching in CH2930		[1, 2]	C-H asymmetric stretching in CH	2938	[1, 2]
C-H symmetric stretching in CH3	2862	[1, 2]	C-H symmetric stretching in CH3	2875	[1, 2]
O=C stretching	1740	[5, 6]	C-H bending	1464	[4]
bidentate COO symmetric stretching	1587	[2, 3]	C-H bending in CH3	1379	[1, 4, 6]
in titanium acetate ligand			C-O asymmetric stretching	1115	[2]
COO symmetric stretching	1427	[3]	C-O	1000-1100	[2]
in titanium acetate ligand			C-O stretching	953	[1]
COO asymmetric stretching	1526	[3]	C-H rocking in CH3	848	[1]
in titanium acetate ligand					
C-H bending in CH3	1360-1390	[1, 4, 6]			
C-H bending in CH2	1278	[6]			
Ti-(O-C)	1123	[4, 2]			
C-O symmetric stretching	1010	[1, 2]			
Ti-O-Ti	930	[7]			
C-H rocking in CH3	851	[1]			
Ti-O asymmetric stretching	667	[6, 3]			

Table S2. Names and processing conditions of samples prepared for XPS measurement.

Sample name	Pre-treatment	Post-treatment
As-deposited	RCA2	No-annealing
RCA2	RCA2	Hotplate@150˚C-1min
HF-dipped	HF-dipped	Hotplate@200˚C-10min
$UV-O_3$	$UV-O_3$	Hotplate@150˚C-1min

REFERENCES

(1) Ahn, K.-H.; Park, Y.-B.; Park, D.-W. Kinetic and Mechanistic Study on the Chemical Vapor Deposition of Titanium Dioxide Thin Films by in Situ FT-IR Using TTIP. *Surf. Coat. Technol.* **2003**, *171* (1), 198–204. https://doi.org/10.1016/S0257-8972(03)00271-8.

(2) Rai, V. R.; Agarwal, S. Surface Reaction Mechanisms during Ozone-Based Atomic Layer Deposition of Titanium Dioxide. *J. Phys. Chem. C* **2008**, *112* (26), 9552–9554. https://doi.org/10.1021/jp8028616.

(3) Goswami, P.; Nath Ganguli, J. Tuning the Band Gap of Mesoporous Zr-Doped TiO 2 for Effective Degradation of Pesticide Quinalphos. *Dalton Trans.* **2013**, *42* (40), 14480–14490. https://doi.org/10.1039/C3DT51891D.

(4) Hua, Z.-L.; Shi, J.-L.; Zhang, L.-X.; Ruan, M.-L.; Yan, J.-N. Formation of Nanosized TiO2 in Mesoporous Silica Thin Films. *Adv. Mater.* **2002**, *14* (11), 830–833. https://doi.org/10.1002/1521-4095(20020605)14:11<830::AID-ADMA830>3.0.CO;2-W.

(5) Pérez-Gallardo, A.; García-Almendárez, B.; Barbosa-Cánovas, G.; Pimentel-González, D.; Reyes-González, L. R.; Regalado, C. Effect of Starch-Beeswax Coatings on Quality Parameters of Blackberries (Rubus Spp.). *J. Food Sci. Technol.* **2015**, *52* (9), 5601–5610. https://doi.org/10.1007/s13197-014-1665-3.

(6) Almeida, A. R.; Moulijn, J. A.; Mul, G. Photocatalytic Oxidation of Cyclohexane over TiO2: Evidence for a Mars−van Krevelen Mechanism. *J. Phys. Chem. C* **2011**, *115* (4), 1330–1338. https://doi.org/10.1021/jp107290r.

(7) Chun, H.; Yizhong, W.; Hongxiao, T. Preparation and Characterization of Surface Bond-Conjugated TiO2/SiO2 and Photocatalysis for Azo Dyes. *Appl. Catal. B Environ.* **2001**, *30* (3), 277–285. https://doi.org/10.1016/S0926-3373(00)00237-X.

(8) He, X.; Zhou, J.; Jin, L.; Long, X.; Wu, H.; Xu, L.; Gong, Y.; Zhou, W. Improved Dielectric Properties of Thermoplastic Polyurethane Elastomer Filled with Core–Shell Structured PDA@TiC Particles. *Materials* **2020**, *13* (15), 3341. https://doi.org/10.3390/ma13153341.

(9) Ribao, P.; Rivero, M. J.; Ortiz, I. TiO2 Structures Doped with Noble Metals and/or Graphene Oxide to Improve the Photocatalytic Degradation of Dichloroacetic Acid. *Environ. Sci. Pollut. Res.* **2017**, *24* (14), 12628–12637. https://doi.org/10.1007/s11356-016-7714-x.

(10) Simon, S. M.; Chandran, A.; George, G.; Sajna, M. S.; Valparambil, P.; Kumi-Barmiah, E.; Jose, G.; Biju, P. R.; Joseph, C.; Unnikrishnan, N. V. Development of Thick Superhydrophilic TiO2–ZrO2 Transparent Coatings Realized through the Inclusion of Poly(Methyl Methacrylate)

and Pluronic-F127. *ACS Omega* **2018**, *3* (11), 14924–14932. https://doi.org/10.1021/acsomega.8b01940.

(11) Gu, Z.; Cui, Z.; Wang, Z.; Sinkou Qin, K.; Asakura, Y.; Hasegawa, T.; Hongo, K.; Maezono, R.; Yin, S. Intrinsic Carbon-Doping Induced Synthesis of Oxygen Vacancies-Mediated TiO2 Nanocrystals: Enhanced Photocatalytic NO Removal Performance and Mechanism. *J. Catal.* **2021**, *393*, 179–189. https://doi.org/10.1016/j.jcat.2020.11.025.

(12) Aghaee, M.; Maydannik, P. S.; Johansson, P.; Kuusipalo, J.; Creatore, M.; Homola, T.; Cameron, D. C. Low Temperature Temporal and Spatial Atomic Layer Deposition of TiO2 Films. *J. Vac. Sci. Technol. A* **2015**, *33* (4), 041512. https://doi.org/10.1116/1.4922588.

(13) McNutt, M. J.; Sah, C. T. Determination of the MOS Oxide Capacitance. *J. Appl. Phys.* **2008**, *46* (9), 3909–3913. https://doi.org/10.1063/1.322138.
